# Enabling Stable Zn Anodes by Molecularly Engineering the Inner Helmholtz Plane with Amphiphilic Dibenzenesulfonimide Additive

**DOI:** 10.1002/advs.202301785

**Published:** 2023-05-18

**Authors:** Jun Yang, Zhiqiang Han, Zhiqiang Wang, Liying Song, Busheng Zhang, Hongming Chen, Xing Li, Woon‐Ming Lau, Dan Zhou

**Affiliations:** ^1^ Beijing Advanced Innovation Center for Materials Genome Engineering & Center for Green Innovation School of Mathematics and Physics University of Science and Technology Beijing Beijing 100083 China; ^2^ Shunde Innovation School University of Science and Technology Beijing Foshan 528000 China; ^3^ School of New Energy and Materials Southwest Petroleum University Chengdu 610500 China; ^4^ School of Chemistry & Chemical Engineering Linyi University Linyi 276005 China

**Keywords:** aqueous batteries, dibenzenesulfonimide, inner Helmholtz plane, stability, Zn‐metal anodes

## Abstract

The notorious dendrite growth and hydrogen evolution reaction (HER) are considered as main barriers that hinder the stability of the Zn‐metal anode. Herein, molecular engineering is conducted to optimize the inner Helmholtz plane with a trace of amphiphilic dibenzenesulfonimide (BBI) in an aqueous electrolyte. Both experimental and computational results reveal that the BBI^−^ binds strongly with Zn^2+^ to form {Zn(BBI)(H_2_O)_4_}^+^ in the electrical double layer and reduces the water supply to the Zn anode. During the electroplating process, {Zn(BBI)(H_2_O)_4_}^+^ is “compressed” to the Zn anode/electrolyte interface by Zn^2+^ flow, and accumulated and adsorbed on the surface of the Zn anode to form a dynamic water‐poor inner Helmholtz plane to inhibit HER. Meanwhile, the{Zn(BBI)(H_2_O)_4_}^+^ on the Zn anode surface possesses an even distribution, delivering uniform Zn^2+^ flow for smooth deposition without Zn dendrite growth. Consequently, the stability of the Zn anode is largely improved with merely 0.02 M BBI^−^ to the common electrolyte of 1 M ZnSO_4_. The assembled Zn||Zn symmetric cell can be cycled for more than 1180 h at 5 mA cm^−2^ and 5 mA h cm^−2^. Besides, the practicability in Zn||NaV_3_O_8_·1.5 H_2_O full cell is evaluated, which suggests efficient storage even under a high mass loading of 12 mg cm^−2^.

## Graphical abstract

Herein, amphiphilic dibenzenesulfonimide (BBI) as an additive is introduced to the aqueous electrolyte to molecularly engineer the inner Helmholtz plane, which suggests great potential in suppressing zinc dendrites and HER for Zn‐metal anode.

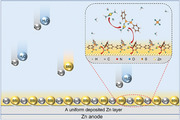

## 1 Introduction

The global mission of embracing carbon neutrality cannot be accomplished without accelerating the replacement of the present primary energy supply mainly derived from fossil fuels with clean electricity principally transferred from solar irradiation and wind, intermittent electricity the usage of which heavily relies on efficient and low‐cost electricity‐storage.^[^ ^1^ ^]^ Currently, over 10 GW of batteries have indeed been installed and over 9 GW of them are lithium‐ion batteries.^[^ ^2^ ^]^ However, the natural abundance of lithium is insufficient to satisfy future market demands and the costs of lithium refinery and recycling are also not low enough for facilitating the mission of carbon neutrality.^[^ ^3^ ^]^ Aqueous zinc batteries are qualified to share the loads in this rapidly changing battery supply chain due to their low cost, high operation‐safety, and acceptably high gravimetric capacity of 820 mAh g^−1^; but the operation‐lifetime of aqueous zinc batteries is still below‐par due to the Zn dendrite growth and hydrogen evolution reaction (HER).^[^ ^4^ ^]^ Although great efforts have been devoted to resolving these technical problems, further harnessing Zn dendrite growth and HER in aqueous zinc batteries very much remains research active.^[^ ^5^ ^]^

Common to all electrochemical interface reactions, the addition of an appropriate surfactant in the electrolytes has been a practical means to harness technical problems similar to those plaguing the practical exploitation of aqueous zinc batteries.^[^ ^6^ ^]^ Indeed, there have been numerous trials of using cationic,^[^ ^7^ ^]^ neutral,^[^ ^8^ ^]^ and anionic surfactants^[^ ^9^ ^]^ to suppress Zn dendrite growth and HER in aqueous zinc batteries. For example, benzyltrimethylammonium chloride (TMBA^+^Cl^−^),^[^ ^7g^ ^]^ a cationic surfactant, facilitates 500 h of cycling under the current‐capacity condition with 5 mA cm^−2^ and 5 mAh cm^−2^ for a standard Zn//Zn configuration. Since cationic surfactants inevitably compete with Zn^2+^ in reference to the transport and supply of cations to the anode surface, neutral surfactants promise performance exceeding that of cationic additives. Indeed, Huang et al.^[^ ^8c^ ^]^ demonstrated that the trace addition of gamma‐butyrolactone (GBL), the adsorption energy of which is 2.9 eV higher than that of water on zinc (4.6 eV), effectively planarizes the plating‐stripping of zinc and suppresses HER. Further, GBL also effectively displaces water in the solvation sheath of Zn^2+^, which, again, suppresses Zn dendrite growth and HER. Specially, the neutral additive of 2‐bis(2‐hydroxyethyl)amino‐2‐(hydroxymethyl)‐1,3‐propanediol effectively chelates Zn^2+^ and yields a good cycling performance of 600 h with a current capacity of 5 mA cm^−2^ to 5 mAh cm^−2^. In this context, anionic surfactants that possess ultrahigh chelating strengths are attractive. Indeed, salts of saccharin (Sac^−^),^[^ ^9b^ ^]^ 3,3′‐dithiodipropane sulfonate (DDS^4−^), and (benzenesulfonyl)benzenesulfonamide^[^ ^9a^ ^]^ show, respectively, the outstanding lifespans of ≈550, 870, and 1000 h for current‐capacity of 5 mA cm^−2^ to 5 mAh cm^−2^. Therefore, anionic surfactants are mainly watched, and expected to improve the cycling stability of Zn anode greatly.

Herein, an anionic surfactant of amphiphilic dibenzenesulfonimide (BBI) as an additive is introduced to the aqueous electrolyte to molecularly engineer the electrode/electrolyte interface, suppressing zinc dendrites and HER for the Zn anode. The experimental and theoretical calculations show that BBI^−^ and Zn^2+^ can form a {Zn(BBI)(H_2_O)_4_}^+^ complex. During the electrodeposition, this complex accumulated and adsorbed at the anode interface to produce a dynamic water‐poor inner Helmholtz plane to inhibit HER. At the same time, the uniform distribution of {Zn(BBI)(H_2_O)_4_}^+^ complex at the interface provides uniform Zn^2+^ flow for flat electrodeposition without Zn dendrite growth. As a result, the assembled Zn||Zn symmetric cell cycles steadily for more than 1730 and 1180 h at current capacities of 2 mA cm^−2^ to 2 mAh cm^−2^ and 5 mA cm^−2^ to 5 mAh cm^−2^, respectively. The Zn||Cu cell achieves a high Coulombic efficiency (CE) of 99.83% at a current capacity of 10 mA cm^−2^ and 5 mAh cm^−2^. Particularly, the practicability is also well verified in Zn||NaV_3_O_8_·1.5 H_2_O (NVOH) full cells and pouch cells with high mass loading.

## 2 Results and Discussion

**Figure 1:**
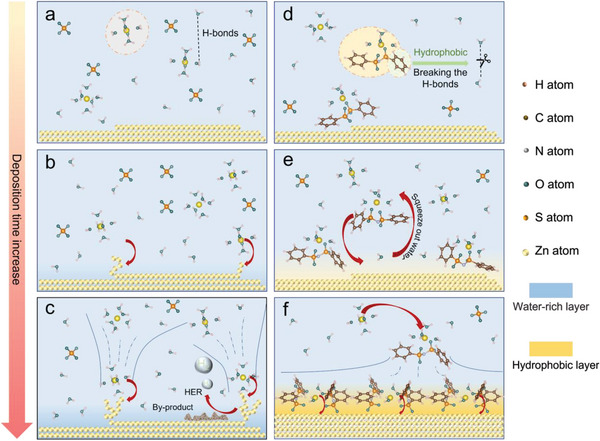
Mechanistic schematics illustrate the suppression of dendrite growth and HER by the trace of BBI^−^. a–c) Zn anode operation with pure 1 M ZnSO_4_; d–f) Zn anode operation with 0.02 M of BBI^−^ in 1 M ZnSO_4_.

The molecular engineering design in the present work is schematically depicted by sequential reaction slap‐shots in **Figure** **1**, which drives the evolution of a 3D optimized electrolyte structure and composition, with mustered BBI^−^ in the anode proximity for the prevention of dendrite growth and HER. Firstly, the inevitable uneven microscopic surface‐topography of a practical anode is portraited by a straight line with steps. When a negative external bias is applied to the anode for zinc deposition, the local electric field strength is particularly high at the edges and corners of these steps. For an electrolyte of a common zinc salt such as ZnSO_4_ (denoted as ZSO), the solvated Zn^2+^, with the known solvated composition of {Zn(H_2_O)_6_}^2+^ which shows six water molecules in its inner solvation sheath and more water molecules in the outer sheath, are transported to the anode proximity where cations are competitively grabbed by the protrusion sites with the highest local electric field‐strength for zinc deposition^[^ ^7b,d^ ^]^(Figure 1a). Dendrites are thus nucleated and catastrophically grown (Figure 1b). The diffusion‐limited factual morphology of these dendrites undesirably facilitates HER, particularly in minute pockets where the diffusion and supply of the solvated Zn^2+^ are blocked. The localized by‐products induced by water hydrolysis, such as the electrically resistive Zn_4_SO_4_(OH)_6_(H_2_O)_n_, further fuel these catastrophic failure mechanisms^[^ ^8a^ ^]^ (Figure 1c). In contrast, the addition of BBI^−^, even just a trace, leads to the effective formation of {Zn(BBI)(H_2_O)_4_}^+^ with a shrinkage in water‐solvation due to the strong binding force in {Zn(BBI)(H_2_O)_4_}^+^ and the presence of hydrophobic molecular moieties in BBI^−^ (Figure 1d). The presence of {Zn(BBI)(H_2_O)_4_}^+^ initiates an intriguing zinc deposition mechanism on the anode surface very different from that of {Zn(H_2_O)_6_}^2+^. The root of such differences is the preferential adsorption of ZnBBI^+^ on microscopically flat facets, with the subsequent zinc deposition following the ZnBBI^+^ adsorption competitively winning over the zinc deposition following the preferential transport of zinc‐containing cations towards edges and corners of surface protrusions (Figure 1e). As such, a surface planarization reaction channel is opened and dendrite growth is suppressed. Further, zinc deposition via {Zn(BBI)(H_2_O)_4_}^+^ adsorption generates BBI^−^ in the inner Helmholtz plane (IHP). The adsorbed BBI^−^ effectively grabs any solvated Zn^2+^ invading the IHP. Further, BBI^−^ released to the outer Helmholtz plane (OHP) cleanses any solvated Zn^2+^ present there. Hence, the dendrite growth channel requiring the presence of {Zn(H_2_O)^2+^ is pinched. When {Zn(H_2_O)^2+^ is depleted in the IHP and OHP, the continuous generation of BBI^−^ in the IHP changes the composition of the IHP from {Zn(BBI)(H_2_O)_4_}^+^ to {Zn(BBI)_2_(H_2_O)_2_} (Figure 1f).

**Figure 2:**
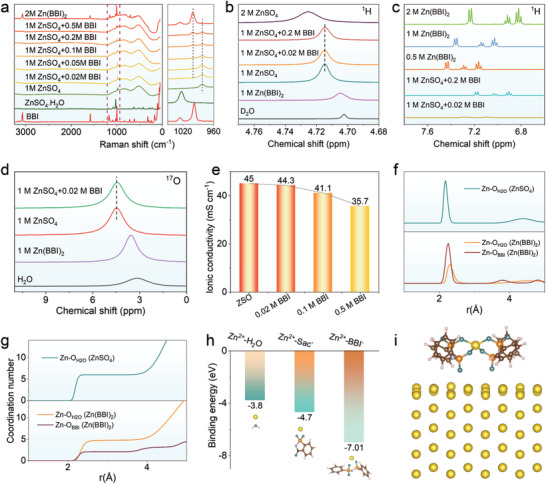
Spectral and computational analyses of Zn(BBI)_2_ solutions and ZnSO_4_ solutions with/without a trace of BBI^−^. a) Raman spectra (the right figure is the enlarged view inside the dotted box in the left figure); b) ^1^H NMR spectra of H_2_O from different electrolytes; c) ^1^H NMR spectra of BBI^−^ from different electrolytes; d) ^17^O NMR spectra of H_2_O from different electrolytes; e) Ionic conductivity of electrolyte with different amounts of BBI^−^, measured at 25 °C; f) Radial distribution functions for Zn‐O (H_2_O) in the ZnSO_4_ electrolyte (in the upper slot), Zn‐O _(H2O)_, and Zn‐O _(BBI)_ in the Zn(BBI)_2_ electrolyte (in the lower slot); g) Coordination number of Zn^2+^ collected from MD simulations in ZnSO_4_ (in the upper slot) and Zn(BBI)_2_ (in the lower slot) electrolytes; h) Binding energy for Zn^2+^ with H_2_O, Sac^−^, and BBI^−^; i) The structure of a Zn(BBI)_2_ molecule adsorbed on Zn (002).

The solvation sheath structures and related coordination characteristics of the electrolytes with/without BBI are detailedly investigated. As seen in the Raman spectra in **Figure** **2**a, the signals at 982.0 and 1155.6 cm^−1^ are associated with the solvation sheath structures of {Zn(H_2_O)_6_}^2+^ and {Zn(BBI)(H_2_O)_4_}^+^,^[^ ^10^ ^]^ respectively. With the addition of BBI in ZSO, the signal at 982.0 cm^−1^ does not indicate any shift, but an independent peak gradually appears at 1155.6 cm^−1^, representing a new solvation sheath formed. In addition, the signal peak of {Zn(H_2_O)_6_}^2+^ at 982.0 cm^−1^ becomes weak, while that of {Zn(BBI)(H_2_O)_4_}^+^ at 1155.6 cm^−1^ tends to strengthen with the increase of BBI. This indicates that BBI^−^ robs Zn^2+^ after entering the solution, promoting the conversion of solvation sheath structure from {Zn(H_2_O)_6_}^2+^ to {Zn(BBI)(H_2_O)_4_}^+^. To further study the two solvation sheath structures, the electrolytes are analyzed by ^1^H NMR. As observed, the chemical shift of ^1^H in pure D_2_O is 4.702 ppm, and it shifts to 4.706 ppm after adding 1 M Zn(BBI)_2_ and to 4.714 ppm after adding 1 M ZnSO_4_. This indicates that there is stronger coordination between Zn^2+^ and water in the ZnSO_4_ electrolyte^[^ ^8f^ ^]^ (Figure 2b). Under different concentrations of the electrolytes with BBI, the H atom on the benzene ring of BBI shows a significant chemical shift, which indicates the strong coordination between BBI^−^ and Zn^2+^ (Figure 2c).

The coordination above is further analyzed by ^17^O NMR. As shown in Figure 2d, the chemical shift of ^17^O in pure H_2_O is 3.181 ppm, and it reveals a slight and sharp increase to 3.563 and 4.481 ppm in 1 M Zn(BBI)_2_ electrolyte and 1 M ZnSO_4_ electrolyte, respectively. The chemical shift of ^17^O in the ZnSO_4_ electrolyte to the lower field indicates that the coordination between Zn^2+^ and ^17^O in H_2_O is stronger. Moreover, in Zn(BBI)_2_ electrolytes with different concentrations, the chemical shift of ^17^O in BBI moves to the low field with the increase of concentration (Figure S1, Supporting Information), which can be attributed to the coordination between Zn^2+^ and ^17^O in BBI, reducing the electron cloud density around the corresponding O atomic nucleus. In sharp contrast, ^17^O in SO_4_^2−^ does not show any chemical shift with the variation of Zn^2+^ concentration. When 0.02 M BBI is added to 1 M ZnSO_4_ (denoted as BBI/ZSO), the chemical shift of ^17^O decreases slightly from 4.481 to 4.419 ppm (Figure 2d). The possible reason is that BBI^−^ robbed Zn^2+^ after entering the electrolyte, which promotes the transformation of {Zn(H_2_O)_6_}^2+^ into {Zn(BBI)(H_2_O)_4_}^+^ solvation sheath structure, which is consistent with the results in Raman spectrum. Furthermore, the ionic conductivity of 1 M ZnSO_4_ suggests a decrease from 45.0 to 44.3 mS cm^−2^ after the addition of 0.02 M of BBI^−^, which further verifies the coupling of Zn^2+^ with BBI^−^ to form {Zn(BBI)(H_2_O)_4_}^+^ (Figure 2e). Figure S2, Supporting Information shows the contact angle results of ZSO and BBI/ZSO electrolytes on the zinc metal surface. The addition of 0.02 M BBI reduces the contact angle from 80.9° to 71.0°, indicating that the hydrophobic benzene ring structure in the BBI molecule breaks the 3D hydrogen bonding network in the aqueous electrolyte. It is well known that the breakage of the hydrogen bond is beneficial to the wettability of electrolytes to electrode materials, enabling the inhibition of dendrite formation and the insertion of Zn^2+^ into cathode materials.

Meanwhile, first‐principle calculations also reveal, as shown in Figure 2f,g and Figure S3, Supporting Information, that Zn^2+^ in 1 M ZnSO_4_ with no BBI^−^ is surrounded by six water molecules in its solvation sheath to form {Zn (H_2_O)_6_}^2+^, with Zn‐O of 2.2 Å. This {Zn (H_2_O)_6_}^2+^ moiety is further weakly solvated by more water molecules with an average Zn‐O distance of 4.5 Å.^[^ ^11^ ^]^ In short, Zn^2+^ is strongly solvated by water and its transport can be considered as a supply of water. This electrolyte condition is drastically changed by the trace addition of BBI^−^, which is driven by the high binding energy of 7.0 eV between Zn^2+^ and BBI^−^, and 3.2 eV more than that between Zn^2+^ and water (Figure 2h). More specifically, in 1 M ZnSO_4_ with 0.02 M BBI^−^, BBI^−^ effectively invades the water‐solvation sheaths of some Zn^2+^ to displace two water molecules to form {Zn(BBI) (H_2_O)_4_}^+^ in the solvation sheath, with Zn‐O_BBI_ of 2.2 Å and Zn‐O_water_ of 2.3 Å (Figure 2g). One of the O atoms in a second BBI^−^ participates in finalizing the solvation sheath, with a Zn‐O_BBI_ separation of 3.7 Å much closer than Zn‐O_water_ of 4.5 Å. The other O atom of this second BBI^−^ is located with a Zn‐O_BBI_ separation of 4.8 Å. This second BBI^−^ cannot get closer to the Zn^2+^ due to steric hindrance. The Zn‐O_water_ separation of {Zn(BBI)(H_2_O)_4_}^+^ is larger than that of {Zn(H_2_O)_6_}^2+^, which implies that stripping water off from {Zn(BBI)(H_2_O)_4_}^+^ is easier than that from {Zn(H_2_O)_6_}^2+^. It is speculated that this water stripping mechanism is active when {Zn(BBI)(H_2_O)_4_}^+^ squeezes into the densely packed IHP of 1 M ZnSO_4_ with 0.02 M BBI^−^. During the anode operation of zinc deposition, {Zn(BBI)(H_2_O)_4_}^+^ is attracted towards the anode surface to upkeep the presence of adsorbed Zn(BBI)^+^on the anode, and subsequently an adsorbed Zn(BBI)^+^ must release its BBI^–^ constituent. As such, this continuous generation of BBI^–^ in the IHP and the replenishment of Zn(BBI)^+^ lead to the formation of adsorbed Zn(BBI)_2_. The computational structure of adsorbed Zn(BBI)_2_ is thus included in Figure 2i which clearly shows a large portion of surface coverage by the four bulky and hydrophobic phenyl rings. The amphiphilic nature of this surface structure facilitates the supply of Zn^2+^ for the anode operation and yet blocks the supply of water for the HER suppression.

**Figure 3:**
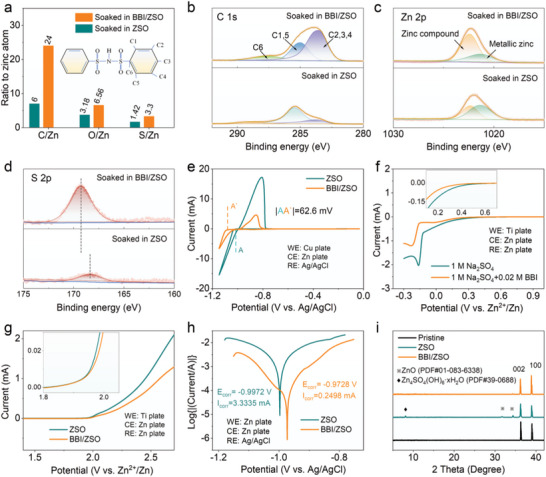
a) Atomic compositions relative to zinc by XPS of a zinc foil soaked in ZSO and BBI/ZSO (The illustration shows the chemical structural formula of BBI); b–d) C 1s, Zn 2p, and S 2p spectra of a zinc foil soaked in ZSO and BBI/ZSO; e) Linear voltammetric scans revealing improvements in redox properties by BBI^−^; f) Suppression of HER by BBI^−^; g) Suppression of oxygen evolution by BBI^−^; h) Linear polarization curves showing the desirable regulation of the anode oxidation by BBI^−^; i) XRD evidence of detrimental zinc oxide formation after 20 cycles under current‐capacity of 1 mA cm^−2^ to 1 mAh cm^−2^ for the case without BBI^−^.

Accordingly, the adsorption of BBI^−^ on the Zn anode is verified by XPS, as shown in **Figure** **3**a–d. Briefly, a comparison of the blow‐dried zinc surface after a soak in 1 M ZnSO_4_ without/with 0.02 M BBI^−^ clearly confirms that while the sample soaked without BBI^−^ shows the typical adventitious oil‐like carbonaceous surface contamination detected by XPS surface‐analysis, with a C/Zn ratio of 6, the sample soaked with BBI^−^ reveals a C/Zn ratio of 24 (Figure 3a). Indeed, the molecule of Zn(BBI)_2_, as depicted in Figure 3b, features a C/Zn ratio of 24. Further, the C 1s spectrum clearly displays the sulfonyl‐C_6_H_5_ signature of BBI^−^, with three types of carbon with decreasing electro‐positivity: one carbon atom directly attaching to sulfonyl, two carbon atoms next to this most electropositive carbon, and three carbon atoms with an electro‐positivity close to that of benzene; the respective spectral peaks are located near 288, 285, and 284 eV. The Zn 2p spectra (Figure 3c) show the presence of an overlayer of zinc compound with a peak near 1023 eV, on the metallic zinc substrate near 1021 eV. For the case of 1 M ZnSO_4_ with 0.02 M BBI^−^, the overlayer is nominally Zn(BBI)_2_, with an overlayer thickness of about 5 nm such that the underlying signals of metallic zinc is very weak (see the explanation in Supporting Information). In comparison, the overlayer on the zinc sample soaked in 1 M ZnSO_4_ is much thinner because the adsorption of ZnSO_4_ is much weaker than that of Zn(BBI)_2_. As such, the intensity of the underlying metallic zinc is even higher than that of the overlayer. The S 2p spectra (Figure 3d) support such an interpretation as the presence of sulfonyl in the case of 1 M ZnSO_4_ with 0.02 M BBI^−^ and the presence of sulfate in the case of 1 M ZnSO_4_ with no BBI^−^ respectively show S 2p at 168.5 and 169.0 eV. Additional evidence of BBI^−^ adsorption and its derivatives in the IHP are included in Figure S4, Supporting Information which comprises the N 1s and O 1s of these samples.

Besides, the composition evolution of the electrical double layer on the Zn anode surface under the presence of the electrolyte of BBI/ZSO, from {Zn(H_2_O)_6_}^2+^ domination to {Zn(BBI)(H_2_O)_4_}^+^ domination, is studied by in situ Raman spectroscopy. As shown in Figure S5, Supporting Information, the Raman peak of {Zn(H_2_O)_6_}^2+^ is located at 980 cm^−1^ and that of {Zn(BBI)(H_2_O)_4_}^+^ is centered at 1000 cm^−1^. Even with the interference of Raman signals from the bulk electrolyte, a considerable drop in the signals of {Zn(H_2_O)_6_}^2+^ is evident in the first few minutes prior to the anode operation entering into a quasi‐steady state. Hence, a trace of BBI^−^ additive is indeed sufficient to drive {Zn(H_2_O)_6_}^2+^out from the electrical double layer by the in situ production of BBI^−^ in the IHP during the anode operation.

In order to study the electrochemical properties of Zn anode in electrolytes with/without BBI, nucleation overpotential, linear polarization experiments, and chronoamperometry (CA) tests are conducted. As seen, the addition of BBI^−^ results in a nucleation overpotential 62.6 mV higher than that of ZSO (Figure 3e) and a reduction in the redox rate versus applied voltage (Figure 3f,g). These changes imply the effective passivation of protrusions on the anode. Larger nucleation overpotential leads to a smaller nucleation radius, which means it is easier to achieve uniform deposition in BBI/ZSO. In addition, Figures S6 and S7, Supporting Information shows SEM images of different electrolytes nucleating on the surface of a Cu foil. Clearly, the electrolyte of BBI/ZSO gives a significantly smoother deposition morphology. SEMs of representative resultant anode surfaces with different electrochemical conditions and with reaction durations as long as several days consistently confirm the reduction of surface artefacts and surface roughness by adding 0.02 M of BBI^−^ to 1 M ZnSO_4_, as well as BBI^−^ to 1 M ZnCl_2_ (Figure S8, Supporting Information and additional descriptions in the Supporting Information). Further, the linear polarization curves shown in Figure 3h indicate that adding BBI^−^ raises the corrosion potential and thus the thermodynamic stability of the anode. The XRD pattern of Zn foil after 20 cycles under the current and capacity of 1 mA cm^−2^ and 1 mAh cm^−2^ in the Zn||Zn symmetric battery is shown in Figure 3i. Two new peaks of ZnO and Zn_4_SO_4_(OH)_6_·*x*H_2_O are found in the zinc foil in the ZSO electrolyte,^[^ ^12^ ^]^ but not in the BBI/ZSO electrolyte. It is further confirmed that BBI can effectively inhibit the side reaction between electrolytes and Zn.

**Figure 4:**
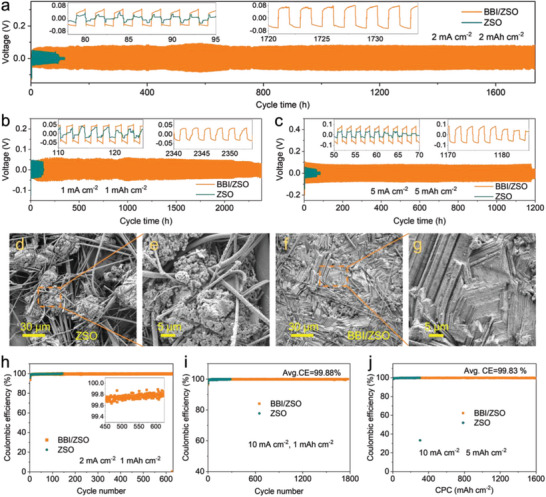
Electrochemical performance improvements by BBI^−^: a–g) Results from Zn||Zn symmetric cells of ZSO and BBI/ZSO; a) Current‐capacity of 2‐2; b) 1 mA cm^−2^ to 1 mAh cm^−2^ and c) 5 mA cm^−2^ to 5 mAh cm^−2^; d,e) SEM of the anode after 20 cycles under 1 mA cm^−2^ to 1 mAh cm^−2^: d,e) In ZSO, showing secondary‐nucleation‐led loose‐morphology; f,g) In BBI/ZSO, showing dense facet‐morphology; h–j) Coulombic efficiencies of Zn||Cu cells in ZSO and BBI/ZSO: h) Current‐capacity of 2 mA cm^−2^ to 1 mAh cm^−2^; i) 10 mA cm^−2^ to 1 mAh cm^−2^; j) 10 mA cm^−2^ to 5 mAh cm^−2^.

The stability and lifespans of the Zn anode are further tested and assessed systematically, with the common configuration of a Zn||Zn symmetric battery. As shown in **Figure** **4**a, a cycle lifespan of only 80 h at the current capacity of 2 mA cm^−2^ to 2 mAh cm^−2^ is recorded without BBI^−^ and it is extended to more than 1730 h with 0.02 M BBI^−^. Similarly, the lifespan at a current capacity of 1 mA cm^−2^ to 1 mAh cm^−2^ without BBI^−^ is merely 110 h but it exceeds 2350 h (Figure 4b) with BBI^−^. Further, at a current capacity of 5 mA cm^−2^ to 5 mAh cm^−2^, the lifespan is 1180 h (cumulative plating capacity of 2.95 Ah cm^−2^, Figure 4c) and at a current capacity of 10 mA cm^−2^ to 10 mAh cm^−2^, it is 190 h (Figure S9, Supporting Information). For those operations with current capacities of 5 mA cm^−2^ to 1 mAh cm^−2^, 10 mA cm^−2^ to 1 mAh cm^−2^, 20 mA cm^−2^ to 1 mAh cm^−2^, and 30 mA cm^−2^ to 1 mAh cm^−2^, the respective lifespans are 3630, 1280, 940, and 470 h (Figure S10, Supporting Information). In addition, this BBI^−^ additive, as shown in Figures S11 and S12, Supporting Information, also significantly improves the lifespans of zinc batteries with aqueous electrolyte systems such as ZnCl_2_ and zinc trifluoromethanesulfonate (Zn(OTf)_2_).

Moreover, the influences of BBI^−^ concentrations are accordingly examined. Briefly for both current capacities of 2 mA cm^−2^ to 2 mAh cm^−2^ (Figure S13, Supporting Information) and 1 mA cm^−2^ to 1 mAh cm^−2^ (Figure S14, Supporting Information) in Zn||Zn symmetrical cell and Zn||Cu half cell (Figure S15, Supporting Information), the BBI^−^ concentration of 0.02 M appears to be optimal. We speculate that insufficient BBI^−^ fails the generation of a dense {Zn(BBI)(H_2_O)_4_}^+^ in the IHP and the blockage of {Zn(H_2_O)_6_}^2+^ to the anode. As for the failure caused by adding too much BBI^−^, we note that adding BBI^−^ to ZSO inevitably reduces the electrolyte conductivity and raises the reaction voltage for the same current density condition. The presence of a high electric field across the electrical double layer increases the risk of leaking {Zn (H_2_O)_6_}^2+^ through the electrical double layer, particularly at some protrusion tips and edges.

The deposition morphology of zinc in Zn||Zn symmetrical cells in different electrolytes after 20 cycles with a current capacity of 1 mA cm^−2^ to 1 mAh cm^−2^ are further examined. Figure S16a,d, Supporting Information are digital photographs of the zinc foils. Clearly, zinc is deposited non‐uniformly in the ZSO electrolyte but uniformly on the whole surface of the BBI^−^/ZSO electrolyte. More specifically, the zinc deposition in ZSO electrolyte is dendritic with a loose morphology (Figure S16b,c, Supporting Information). In comparison, the zinc deposition in BBI^−^/ZSO electrolyte has a bright metallic luster (Figure S16e,f, Supporting Information), which indicates that a flat zinc deposition is produced, with few light scattering loss and dark patches like those in Figure S16b,c, Supporting Information. Similarly, SEM images also show that the zinc deposition in the ZSO electrolyte features uneven dendritic (Figure 4d) and a loose porous microstructure (Figure 4e), whereas the zinc deposition in the BBI^−^/ZSO electrolyte is smooth (Figure 4f), with planarized zinc deposition (Figure 4g). Electrochemical impedance spectroscopy (EIS) is a powerful tool for studying dendrites and side reactions.^[^ ^13^ ^]^ Figure S17, Supporting Information shows the EIS curves of Zn||Zn symmetric cell before and after cycling. There is no sign of a short circuit or sudden decrease of *R_ct_* during the whole cycle, which confirms that the Zn||Zn cell with BBI/ZSO delivers good cycling stability. This result strongly supports the fact that BBI additives enable uniform Zn electrodeposition and inhibit side reactions.

Desirably, the BBI^−^ additive also performs well in Zn||Cu asymmetric cells. Figure 4h shows the Coulombic efficiency (CE) of different electrolytes for the current capacity of 2 mA cm^−2^ to 1 mAh cm^−2^. The battery with ZSO fails after only 140 deposition/stripping cycles with a sudden short circuit but operates for more than 630 cycles with an average CE of 99.7%. For current‐capacity of 10 mA cm^−2^ to 1 mAh cm^−2^ and 20 mA cm^−2^ to 1 mAh cm^−2^, the respective lifespans and CEs are > 1700 cycles (600 h) and 99.88%, >1100 cycles (300 h) and 99.90%.

In reference to the benchmarking measurements of cumulative plating capacity (CPC),^[^ ^7e^ ^]^ a Zn||Cu half‐cell in ZSO is tested which shows a CPC of > 1600 mAh cm^−2^ with 0.02 M BBI^−^ and only 245 mAh cm^−2^ with no BBI^−^, for current‐capacity of 10 mA cm^−2^ to 5 mAh cm^−2^ (Figure 4j). Further increasing the current capacity to 20 mA cm^−2^ to 10 mAh cm^−2^, the CPC with BBI^−^ is 800 and 90 mAh cm^−2^ in the absence of BBI^−^ (Figures S18 and S19, Supporting Information). In fact, the tests of Zn||Cu half cells show that the BBI^−^ additive is effective in a wide range of currents and capacities. As shown in Table S1, Supporting Information, a systematic comparison of cycling stability for Zn||Zn symmetric cells and CE for Zn||Cu asymmetric cells between this work and the recently published reports using different additives and electrolytes to optimize aqueous zinc batteries clearly indicates that the present work with BBI^−^ is outstandingly competitive.

**Figure 5:**
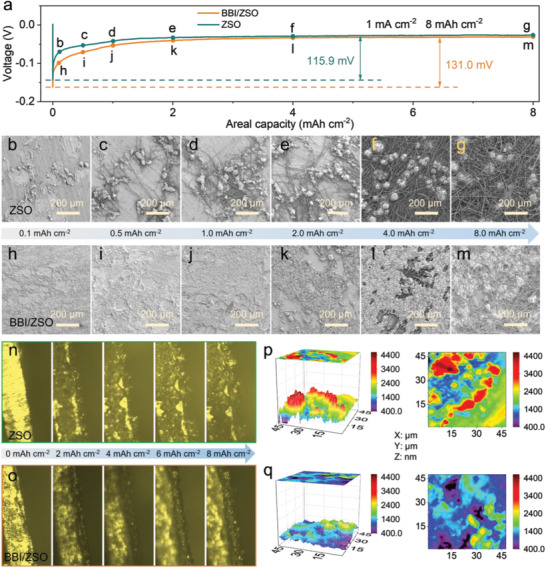
a) Capacity voltage profiles of the first nucleation of Zn in Zn||Zn symmetric cell at the current and capacity of 1 mA cm^−2^ to 8 mA h cm^−2^; b–m) SEM images of deposition morphology of Zn with constant current but different capacities in electrolyte b—g) without or h—m) with BBI; n,o) In situ microscopy images of the Zn plating process in n) ZSO electrolyte and o) BBI/ZSO electrolyte; p,q) AFM of Zn||Zn symmetric cells after 20 cycles at 1 mA cm^−2^ to 1 mA h cm^−2^ in p) ZSO electrolyte and q) BBI/ZSO electrolyte.

Additionally, in order to better understand the real situation of Zn nucleation on Zn substrate with/without BBI additive's electrolytes, the morphology of Zn deposition with different capacities under constant current is also observed. **Figure** **5**a shows the voltage characteristics of zinc deposited on zinc substrate by different electrolytes. The zinc deposited in the BBI/ZSO electrolyte has a nucleation overvoltage of 131.0 mV, slightly higher than 115.9 mV in the ZSO electrolyte, which is consistent with the test results in Zn||Cu cells (Figure 3f). The higher overpotential in the BBI/ZSO electrolyte may be due to the greater binding force between BBI and Zn^2+^, which improves the energy barrier of Zn deposition and provides a greater driving force for Zn nucleation to produce flat deposition. As shown in Figure 5b–g, Zn deposits of different capacities in the ZSO electrolyte all show obviously aggregated particles, which should be related to the charge aggregation at the electrolyte/electrode interface. In sharp contrast, as shown in Figure 5h,i, the BBI/ZSO electrolyte has a uniform and flat deposition morphology, which should be attributed to the strong binding of BBI^−^ to Zn^2+^ to generate greater desolvation barrier, which promotes the Zn^2+^ uniform distribution at the interface, thus inhibiting the emergence of dendrites. Furthermore, the deposition morphology of 4 mA h cm^−2^ Zn deposited at different currents in different electrolytes is also briefly studied. At all current densities, the BBI/ZSO electrolyte exhibits a larger nucleation overpotential than that of the ZSO electrolyte (Figure S20a–d, Supporting Information). As shown in Figure S20e–h, Supporting Information, the ZSO electrolyte generates granular zinc deposition with a loose porous structure under the current density of 1–4 mA cm^−2^. With the increase of current density, it tends to form relatively compact and uniformly distributed smaller particles. This should be attributed to the fact that the deposition of metal zinc gradually changes from being controlled by surface energy to being controlled by ion diffusion in electrolytes with the acceleration of galvanizing rate.^[^ ^14^ ^]^ As expected, BBI/ZSO electrolyte has formed uniform and flat zinc deposits with closely stacked layer structures at all current densities (Figure S20i–l, Supporting Information). Moreover, the in situ microscope system is used to conduct real‐time optical observation of Zn deposition in different electrolytes. As shown in Figure 5n, zinc deposition in ZSO electrolyte has a small number of isolated deposition sites at a deposition capacity of 2 mA h cm^−2^. These metal cores gradually grow to form sharp dendrites in the subsequent deposition process. However, the Zn deposition in BBI/ZSO electrolyte is evenly distributed and flat, even if the deposition capacity reaches 8 mA h cm^−2^ (Figure 5o). The deposition morphology of Zn in different electrolytes is further analyzed by an atomic force microscope (AFM). As shown in Figure 5p, the deposition of Zn in the ZSO electrolyte is sharper dendritic, while the deposition of zinc in the BBI/ZSO electrolyte is more flat (Figure 5q).

**Figure 6:**
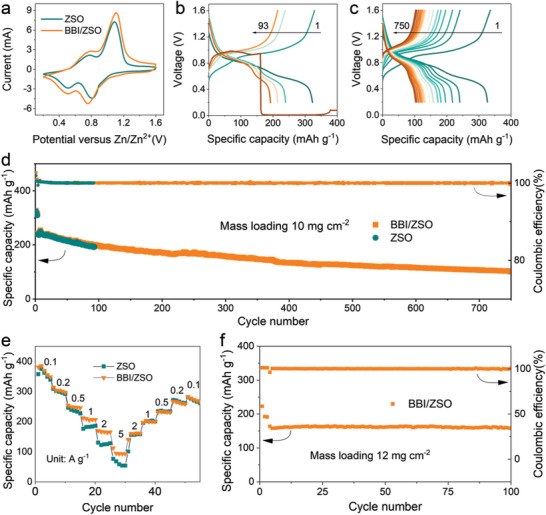
a) Cyclic voltammetric scans of different electrolytes in Zn||NVOH cells; b,c) Charge/discharge profiles of the Zn||NVOH full cells in ZSO and BBI/ZSO; d) Cycling performance of Zn||NVOH full cells in ZSO and BBI/ZSO under the current density of 0.5 A g^−1^; e) Rate capabilities of Zn||NVOH full cells at current densities from 0.1 to 5 A g^−1^; f) Cycling performance of a Zn||NVOH pouch cell under the current density of 0.4 A g^−1^.

To verify the reliability of BBI^−^ additive in practical applications, standard Zn||NVOH full cells with high mass loading are assembled and tested. Figures S21 and S22, Supporting Information show the XRD pattern and SEM images of the synthetic NVOH, which are consistent with the previous report.^[^ ^15^ ^]^ As shown in **Figure** **6**a, the cyclic voltammetry (CV) measurements confirm that BBI^−^ does not affect the oxidation and reduction processes of the NVOH cathode. Figure 6b,c show the charge/discharge diagrams of Zn||NVOH with and without BBI^−^, respectively. Clearly, the Zn||NVOH cell without BBI^−^ fails after 92 cycles. Figure 6d shows the long‐cycle performance tests of ZSO and BBI/ZSO at the current density of 0.5 A g^−1^. The Zn||NVOH full cell with ZSO shows a fast capacity reduction in the cycling operation and fails after 93 cycles. In comparison, the lifespan increases by the addition of 0.2 M BBI^−^ to > 750 cycles with a high CE of 99.9%. As shown in Figure 6e, the high charge and discharge rates of Zn||NVOH cells with BBI^−^ also outperforms other electrolyte formulations. Further, the good performance of the pouch cell with BBI/ZSO, with a capacity retention ratio of 99.5% after 100 cycles, also supports the merit of adding 0.02 M BBI^−^ to 1 M ZnSO_4_ (Figure 6f).

## 3 Conclusion

In summary, a stable Zn anode with suppressed dendrite growth and HER is realized by molecularly engineering the inner Helmholtz plane with a trace addition of 0.02 M BBI^−^ into 1 M ZnSO_4_. It is found that the BBI^−^ binds strongly with Zn^2+^ to form {Zn(BBI)(H_2_O)_4_}^+^ in the electrical double layer and reduces the water supply to the Zn anode. During the electroplating, {Zn(BBI)(H_2_O)_4_}^+^ is “compressed” to the Zn anode/electrolyte interface by Zn^2+^ flow, yielding a dynamic water‐poor inner Helmholtz plane to inhibit HER. At the same time, the{Zn(BBI)(H_2_O)_4_}^+^ at the interface is evenly distributed, which can promote uniform Zn^2+^ flow for the formation of a smooth deposition morphology without Zn dendrite growth. Consequently, a stable Zn anode for aqueous batteries is achieved. The assembled Zn||Zn symmetric cell can be cycled for more than 1180 h at 5 mA cm^−2^ and 5 mA h cm^−2^. The Zn||Cu cell delivers a high CE of 99.83% at a current density of 10 mA cm^−2^. Particularly, typical Zn||NVOH full cell suggests promising practical potential even under high mass loading. This work offers new insights into the realization of stable Zn anodes by molecular engineering with a trace of amphiphilic additive in the electrolytes for aqueous batteries.

## 4 Experimental Section

### Materials

Zinc sulfate monohydrate (99% purity, ZnSO_4_·H_2_O), sodium chloride (99.8% purity, NaCl), deuterium oxide ((99.9% purity, D_2_O), dibenzenesulfonimide (98% purity, BBI), and vanadium pentoxide (99% purity, V_2_O_5_) were purchased from Energy Chemical Co., Ltd. Zinc trifluoromethanesulfonate (98% purity, Zn(OTf)_2_) was purchased from Macklin company. Zinc foil (99.99% purity, 0.2 mm thickness), titanium foil (0.03 mm thickness), copper foil (99.99% purity, 0.05 mm thickness), Whatman glass fiber membrane (GF/D 1823‐055), polyvinylidene fluoride (PVDF), N‐methyl pyrrolidone (99% purity, NMP), carbon black, and CR2032 button battery case were purchased from Guangdong Canrd New Energy Technology Co., Ltd.

### Synthesis of NaV_3_O_8_·1.5 H_2_O (NVOH) Nanobelts and Electrolytes

3 g V_2_O_5_ was added into 90 mL 2 M NaCl aqueous solution and stirred at 25 °C for 72 h.^[^ ^15^ ^]^ The obtained reddish‐brown precipitate was washed several times with ethanol and deionized water, and the resultant reddish‐brown product was transferred to a 60 °C oven for drying. Add 1 M of ZnSO_4_·H_2_O and 0.02 M of BBI into 1 L of ultra‐pure water and stir to obtain BBI/ZSO electrolyte.

### Electrochemical Measurements

The Tafel curve was tested at Corrtest Electrochemical Workstation (CS350M) with a three‐electrode system, and the scanning rate was 0.5 mV s^−1^. Zn was used as the working electrode/counter electrode, and the saturated Ag/AgCl electrode was used as the reference electrode. The linear sweep voltammetry (LSV) curve was tested in the CR2032 coin‐type cells using the two‐electrode method, with Ti foil as the working electrode and Zn as the counter electrode/reference electrode, and the scanning rate is 0.5 mV s^−1^. When testing the hydrogen evolution potential, the ZnSO_4_ in the electrolyte was replaced with Na_2_SO_4_ to eliminate the influence of electrodeposition.

Zn||Zn, Zn||Cu and full cells were tested using Coin‐type CR2302 with a Galvanostatic BTS battery tester (NEWARE Electronics Co., Ltd.) under a voltage range of 0.2–1.6 V versus Zn/Zn^2+^. The cells were first charged and discharged at 0.1 A g^−1^ for three cycles, followed by a regiment of charging at 0.5 A g^−1^. All the cells were assembled in the ambient atmosphere. Specific parameters of accessories in cell assembly are as follows: copper foil (19 mm diameter), zinc sheet (14 mm diameter), glass fiber separator (19 mm diameter), aqueous electrolyte (90 µL), steel sheet (1 mm thickness). The mixture of NVOH: carbon black: PVDF with a mass ratio of 7:2:1 was dispersed in NMP. The slurry was then scraped onto the titanium foil surface. After drying, the cathode was obtained, and the active mass loading was 10–12 mg cm^−2^.

### Material Characterizations

The crystallographic data of samples were obtained by a Bruker D2 Phaser X‐Ray Diffractometer (XRD). The Fourier transform infrared spectroscopy (FTIR) mapping was performed with the Nicolet iS50 FT‐IR Instrument. Raman spectroscopy (DXR3x) was collected with a 532 nm laser. ^1^H spectra were obtained by a Bruker Advance 300. The microscopic morphology was observed by scanning electron microscope (ZEISS Sigma 300). Dendrite growth was observed in situ with Nreeohy metallographic microscope. The ionic conductivity was tested by Shanghai Thundermagnetic DDBJ‐350 portable conductivity tester.

### Calculation Methods

Gromacs 2018 was employed to perform the molecular dynamics simulation, with electrolyte molar ratios taken from those used in the experimental work. All molecules’ topology files and bonded and Lennard–Jones parameters were generated by using the Sobtop package.^[^ ^16^ ^]^ The atomic charges involved in the analyses were evaluated by fitting the molecular RESP by Multiwfn based on the highly effective algorithm proposed.^[^ ^17^ ^]^ The simulation procedure consisted of energy minimization using the steepest descent method followed by a 1 ns equilibration step using a Berendsen barostat and a 30 ns production run using a Parrinello–Rahman barostat, both at a reference pressure of 1 bar with a timestep of 1 ns. A Nosé–Hoover thermostat was used throughout with a reference temperature of 300 K. The particle mesh Ewald method was used to calculate electrostatic interactions. Periodic boundary conditions were applied in all directions. Bonds with hydrogen atoms were constrained. The final 20 ns of the production run was used for the analysis. In addition, the calculations were carried out with the implicit universal solvation model based on solute electron density (SMD). The molecular geometries (Zn^2+^·6H_2_O, H_2_O, ZnSO_4,_ and BBI) were optimized and calculated by DFT by using the Gaussian 09 package at the B3LYP/6‐311G + (d, p) level.

## Conflict of Interest

The authors declare no conflict of interest.

## Supporting information

Supporting Information

## Data Availability

The data that support the findings of this study are available from the corresponding author upon reasonable request.

## References

[1] a) L. Yuan , J. Hao , C.‐C. Kao , C. Wu , H.‐K. Liu , S.‐X. Dou , S.‐Z. Qiao , Energy Environ. Sci. 2021, 14, 5669;

[2] a) Y. Xu , G. Zhang , J. Liu , J. Zhang , X. Wang , X. Pu , J. Wang , C. Yan , Y. Cao , H. Yang , W. Li , X. Li , Energy Environ. Mater. 2022, 10.1002/eem2.12575e12575;

[3] a) D. Han , C. Cui , K. Zhang , Z. Wang , J. Gao , Y. Guo , Z. Zhang , S. Wu , L. Yin , Z. Weng , F. Kang , Q.‐H. Yang , Nat. Sustainability 2022, 5, 205;

[4] a) Z. Wang , Y. Li , J. Wang , R. Ji , H. Yuan , Y. Wang , H. Wang , Carbon Energy 2022, 4, 411;

[5] a) X. Zhang , J. P. Hu , N. Fu , W. B. Zhou , B. Liu , Q. Deng , X. W. Wu , InfoMat 2022, 4, 12306;

[6] a) Z. Wang , H. Chen , H. Wang , W. Huang , H. Li , F. Pan , ACS Energy Lett. 2022, 7, 4168;

[7] a) A. Bayaguud , X. Luo , Y. Fu , C. Zhu , ACS Energy Lett. 2020, 5, 3012;

[8] a) H. Lu , X. Zhang , M. Luo , K. Cao , Y. Lu , B. B. Xu , H. Pan , K. Tao , Y. Jiang , Adv. Funct. Mater. 2021, 31, 2103514;

[9] a) H. Du , Y. Dong , Q. J. Li , R. Zhao , X. Qi , W. H. Kan , L. Suo , L. Qie , J. Li , Y. Huang , Adv. Mater. 2023, 10.1002/adma.202210055e2210055; 36637812

[10] J. Cao , D. Zhang , Y. Yue , R. Chanajaree , S. Wang , J. Han , X. Zhang , J. Qin , Y. Huang , Nano Energy 2022, 93, 106839.

[11] M. Han , J. Huang , X. Xie , T. C. Li , J. Huang , S. Liang , J. Zhou , H. J. Fan , Adv. Funct. Mater. 2022, 32, 2110957.

[12] a) X. Zeng , K. Xie , S. Liu , S. Zhang , J. Hao , J. Liu , W. K. Pang , J. Liu , P. Rao , Q. Wang , J. Mao , Z. Guo , Energy Environ. Sci. 2021, 14, 5947;

[13] a) Q. Li , A. Chen , D. Wang , Z. Pei , C. Zhi , Joule 2022, 6, 273;

[14] Z. Cai , J. Wang , Z. Lu , R. Zhan , Y. Ou , L. Wang , M. Dahbi , J. Alami , J. Lu , K. Amine , Y. Sun , Angew. Chem., Int. Ed. 2022, 61, 202116560. 10.1002/anie.202116560 35088500

[15] X. Rui , Y. Tang , O. I. Malyi , A. Gusak , Y. Zhang , Z. Niu , H. T. Tan , C. Persson , X. Chen , Z. Chen , Q. Yan , Nano Energy 2016, 22, 583.

[16] T. Lu , Sobtop, 1.0(dev3.1), http://sobereva.com/soft/Sobtop (accessed on August 2022).

[17] J. Zhang , T. Lu , Phys. Chem. Chem. Phys. 2021, 23, 20323. 34486612 10.1039/d1cp02805g

