# Supplementary material for: Enabling Stable Zn Anodes by Molecularly Engineering the Inner Helmholtz Plane with Amphiphilic Dibenzenesulfonimide Additive
Source: Adv Sci (Weinh). 2023 May 18;10(22):2301785. doi: 10.1002/advs.202301785 (PMC10401170; doi:10.1002/advs.202301785)
Supplement: Supplementary file 1 — Supporting Information [file ADVS-10-2301785-s001.pdf]

ADVANCED SCIENCE
Open Access

Supporting Information

for *Adv. Sci.*, DOI 10.1002/advs.202301785

Enabling Stable Zn Anodes by Molecularly Engineering the Inner Helmholtz Plane with Amphiphilic Dibenzenesulfonimide Additive

Jun Yang, Zhiqiang Han, Zhiqiang Wang, Liying Song, Busheng Zhang, Hongming Chen, Xing Li, Woon-Ming Lau* and Dan Zhou**

Supporting Information

Enabling Stable Zn Anodes by Molecularly Engineering the Inner Helmholtz Plane with Amphiphilic Dibenzenesulfonimide Additive

Jun Yang[1,2#], Zhiqiang Han[1,2#], Zhiqiang Wang[1,2], Liying Song[1,2], Busheng Zhang[1,2], Hongming Chen[1,2], Xing Li[4*], Woon-Ming Lau[1,2,3*], Dan Zhou[1,2*]

[1] Beijing Advanced Innovation Center for Materials Genome Engineering & Center for Green Innovation, School of Mathematics and Physics, University of Science and Technology Beijing, Beijing 100083, China

[2] Shunde Innovation School, University of Science and Technology Beijing, Foshan, Guangdong 528000, China

[3] School of Chemistry & Chemical Engineering, Linyi University, Linyi, China 276005.

[4] School of New Energy and Materials, Southwest Petroleum University, Chengdu 610500, China.

Jun Yang and Zhiqiang Han contributed equally to this work.

* Corresponding authors: lixing@swpu.edu.cn (X. Li); leolau@lyu.edu.cn (W. M. Lau); zhoudan@ustb.edu.cn (D. Zhou)

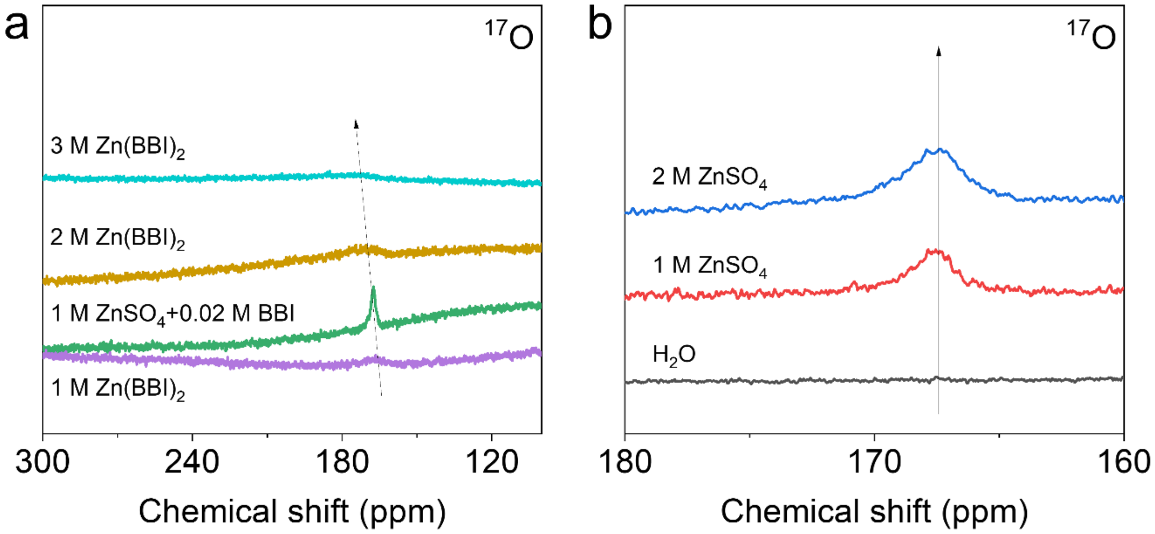

Figure S1. ^{17}O NMR spectra of BBI (a) and SO_4^{2-} (b) from different electrolytes.

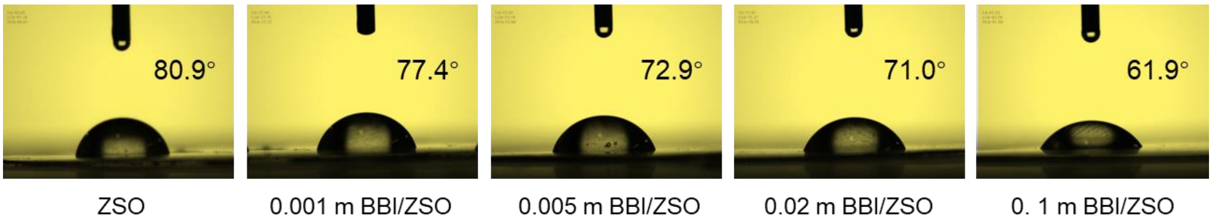

Figure S2. Contact angle measurement of different electrolytes on zinc substrates.

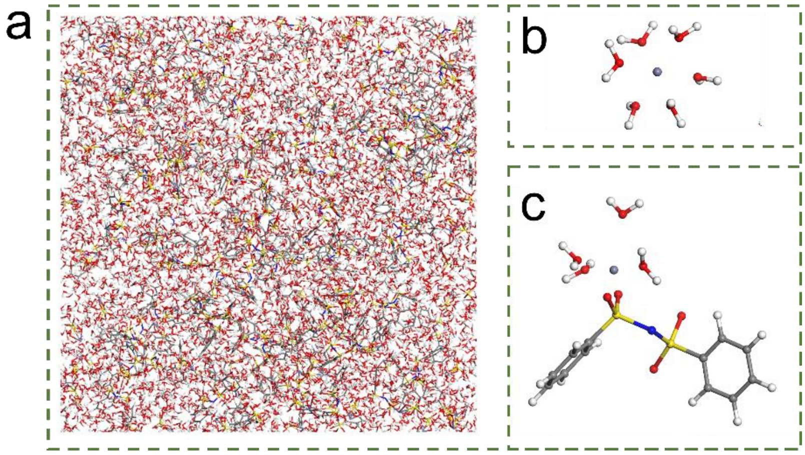

Figure S3. a. 3D snapshot obtained by MD simulations and representative Zn^{2+}-inner solvation structure of $ZnSO_4$ (b) and $Zn(BBI)_2$ (c) electrolyte.

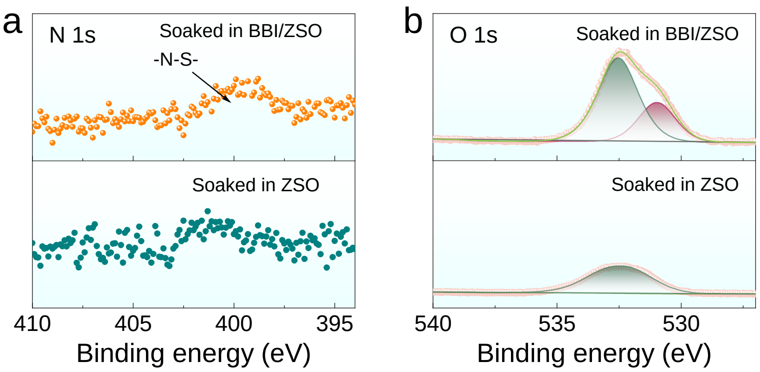

Figure S4. X-ray photoelectron spectroscopy of zinc foil surface soaked in electrolyte w/wo BBI; N 1s (a) and O 1s (b) spectra (In XPS analysis of an overlayer structure, overlayer thickness can be estimated by multiplying the photoelectron inelastic mean free path in the overlayer with ln(1+(detected photoemission from the overlayer)/ detected photoemission from the underlayer). In accord to the work by Powell et al.[1], the inelastic mean free path for the photoelectrons of Zn 2p in the present study is about 1.0 nm. Hence, the overlayer thickness is 1.0nm *5.3 = 5.3nm).

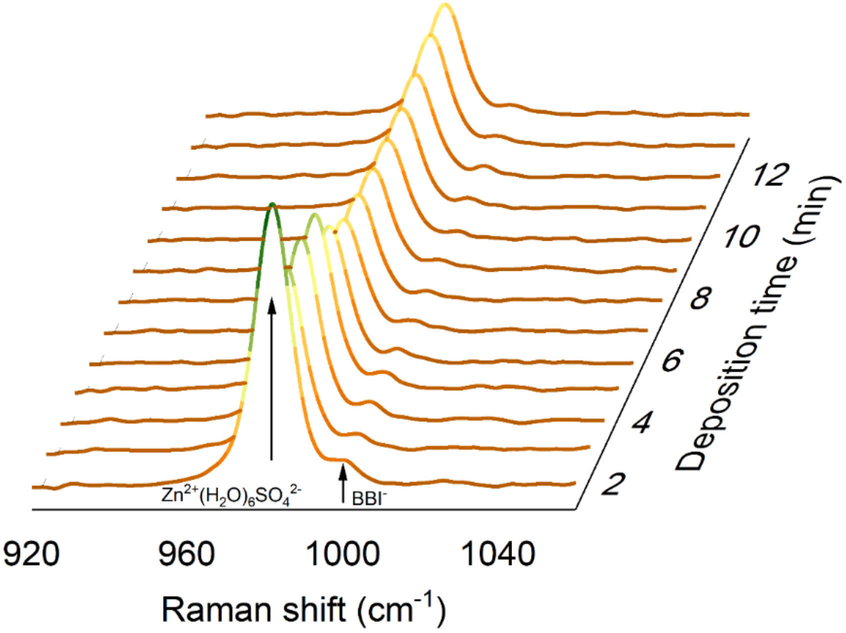

Figure S5. *In-situ* Raman spectrum of Zn electrodeposition in BBI/ZSO electrolyte.

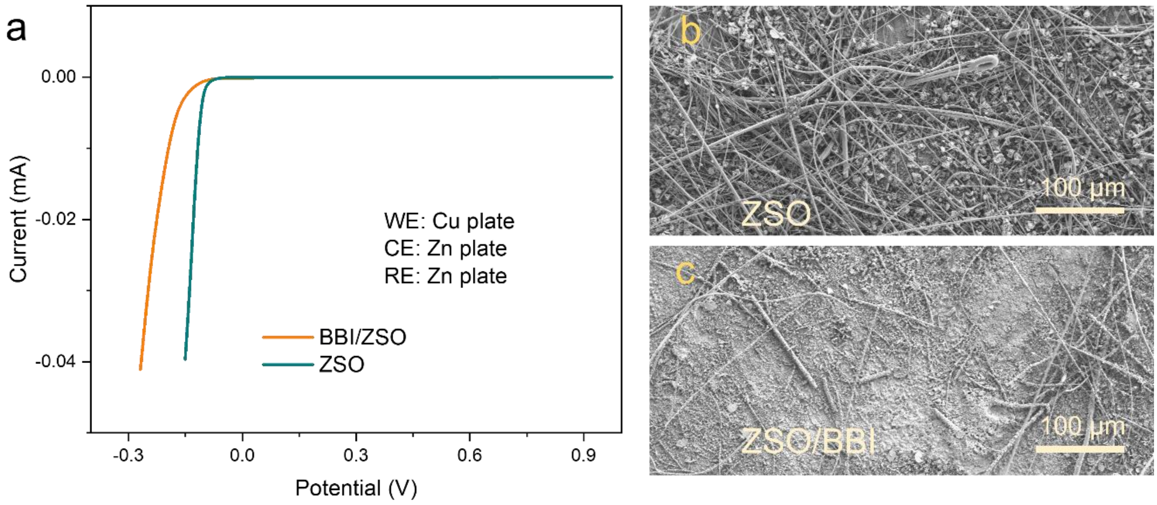

Figure S6. Nucleation overpotential test (a) and deposition morphology of Zn in different electrolytes on Cu foil surface. b. ZSO, c. BBI/ZSO.

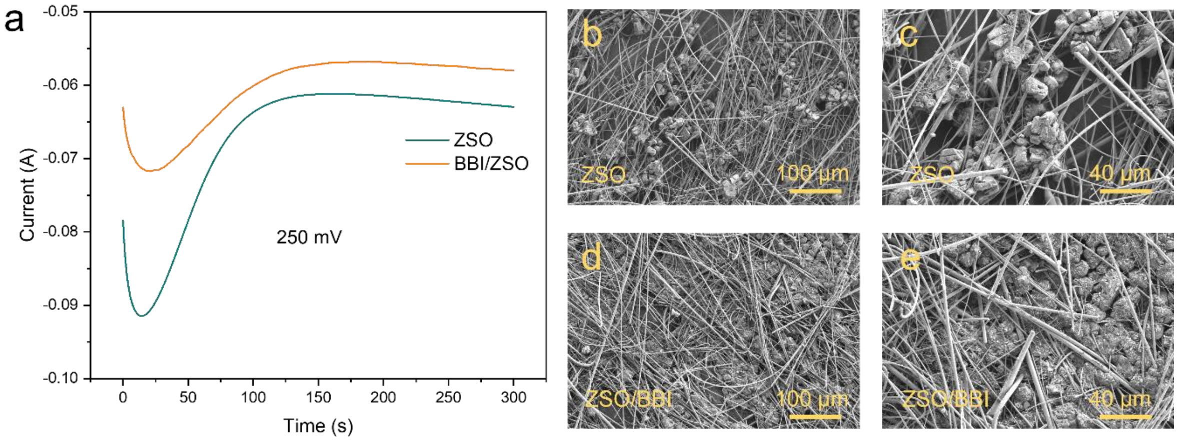

Figure S7. Chronoamperometry measurements in Zn||Zn symmetric cells (a) and corresponding SEM images of deposition morphology of Zn on Zn foil surface in different electrolytes. b-c. ZSO, d-e. BBI/ZSO.

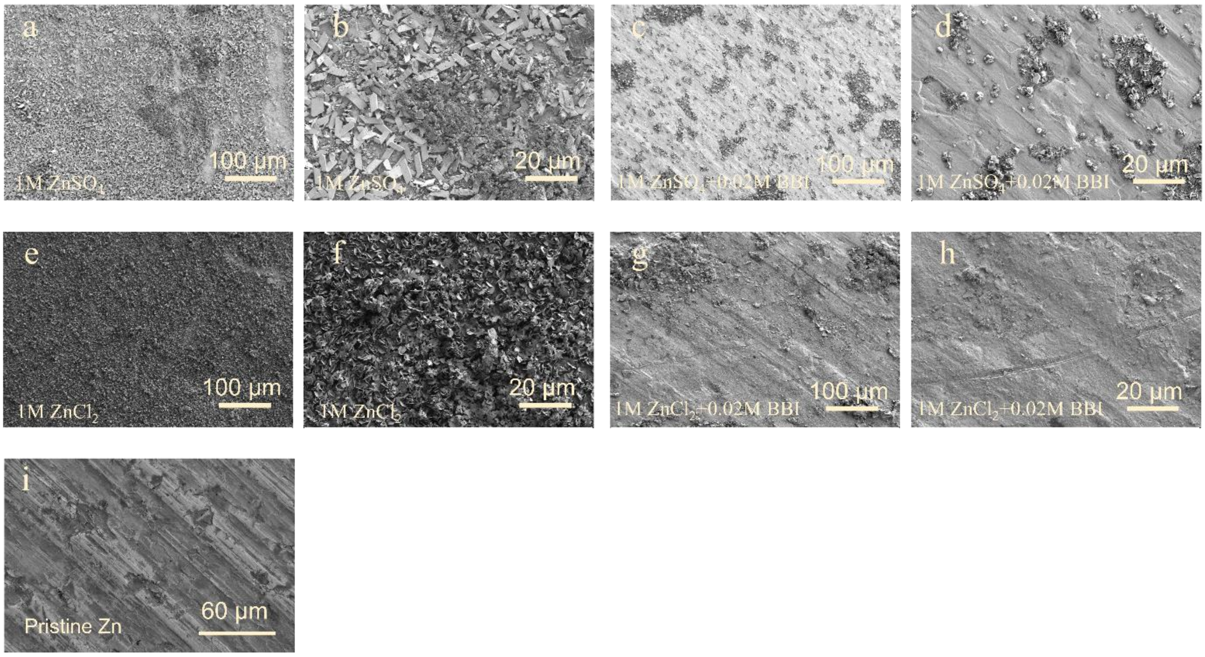

Figure S8. SEM images of zinc foil immersed in different electrolytes for 7 days, a-b. ZSO, c-d. BBI/ZSO, e-f. 1 M $ZnCl_2$, g-h. 1 M $ZnCl_2$+0.02 M BBI. i. Pristine Zn foil (After 7 days of immersion experiments in different electrolytes, the surface of Zn foil in 1 m $ZnSO_4$ or 1 m Zinc chloride ($ZnCl_2$) aqueous electrolyte has thick flake species, whereas only a small amount of flake species appears on the surface of Zn foil in the electrolyte with BBI added).

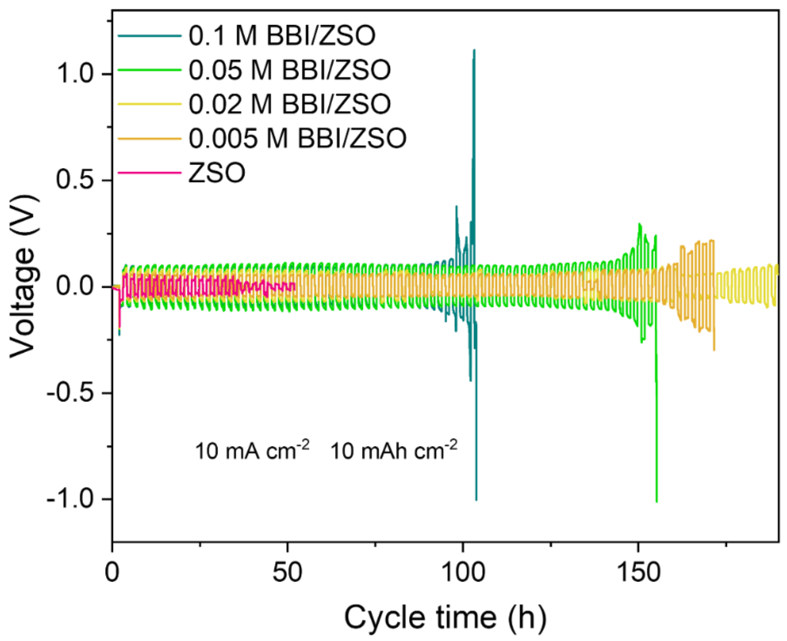

Figure S9. Long-term galvanostatic deposition/stripping of Zn in Zn||Zn symmetric cells in 1 M $ZnSO_4$ and 1 M $ZnSO_4$+0.02 M BBI electrolytes at 10 mA cm^{-2}@10 mA h cm^{-2}.

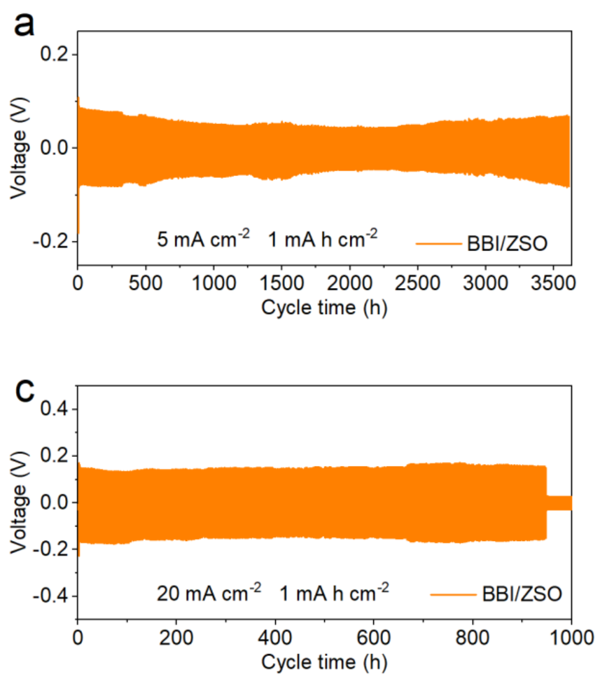

Figure S10. Long-term galvanostatic deposition/stripping of Zn in Zn||Zn symmetric cells in 1 m $ZnSO_4$+0.02 m BBI electrolyte at (a) 5 mA cm^{-2}@1 mA h cm^{-2}, (b) 10 mA cm^{-2}@1 mA h cm^{-2}, (c) 20 mA cm^{-2}@1 mA h cm^{-2}, (d) 30 mA cm^{-2}@1 mA h cm^{-2}.

Significantly longer cycle life of Zn//Zn symmetric cell was achieved at a lower deposition capacity under the same current density, or at the same deposition capacity under a higher current. This should be attributed to the following reasons:

1) With the same thickness of the separator and constant current density, a higher deposition capacity will produce relatively larger dendrites, which can rapidly accumulated and penetrated the separator. A lower deposition capacity will produce smaller dendrite volumes, which take longer to accumulate enough dendrites and pierce the separator to cause short-circuit.

2) When the deposition capacity is the same of 1mAh cm^{-2}, an appropriate increase in current density can significantly improve the cycle time, such as 5mA cm^{-2}. The recent report (*Angew. Chem. Int. Ed. Engl. 2022, 61 (14), e202116560;* ***Ref. 14***) and this experiment have both shown that a higher current density can produce a smoother deposition morphology (**Figure S20**), due to the fact that a higher current can suppress the chaotic motion of zinc ions on the anode surface during deposition and suppress dendrites.

The two factors may be responsible for the fact that the cycle time of the Zn||Zn symmetric cell under the condition of 5mA cm^{-2}@1mAh cm^{-2} is much longer than that under the condition of 5mA cm^{-2}@5mAh cm^{-2} or 1mA cm^{-2}@1mAh cm^{-2}.

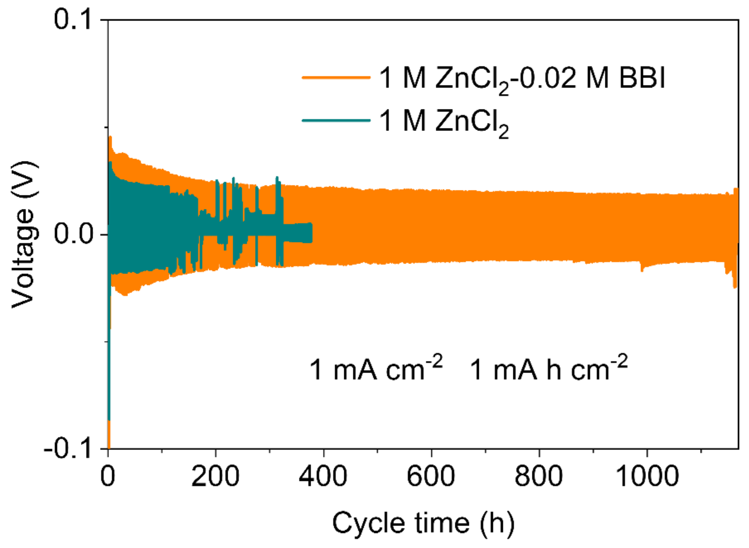

Figure S11. Long-term galvanostatic deposition/stripping of Zn in Zn||Zn symmetric cells in 1 M $ZnCl_2$ and 1 M $ZnCl_2$+0.02 M BBI electrolytes at 1 mA cm^{-2}@1 mA h cm^{-2}.

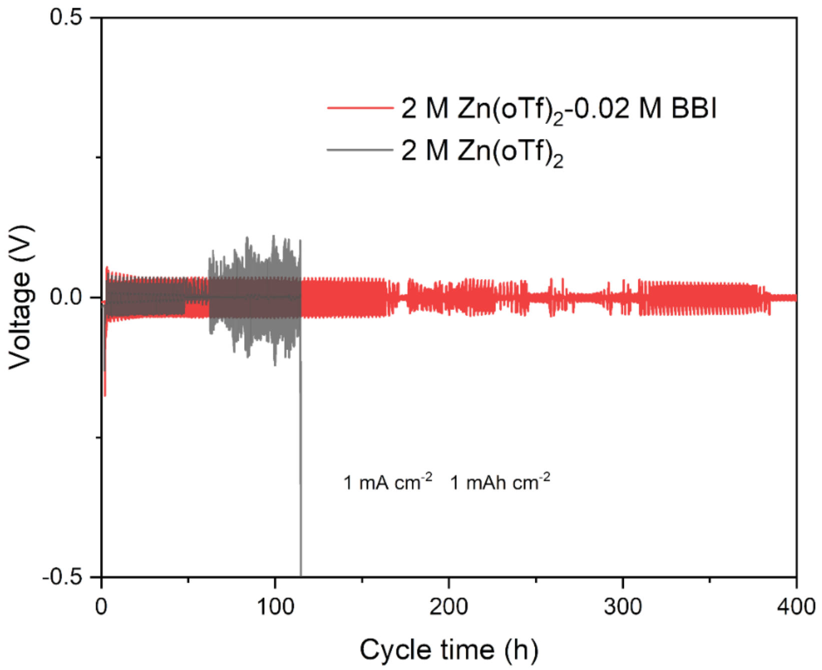

Figure S12. Long-term galvanostatic deposition/stripping of Zn in Zn||Zn symmetric cells in 2 M $Zn(OTf)_2$ and 2 M $Zn(OTf)_2$+0.02 M BBI electrolytes at 1 mA cm^{-2}@1 mA h cm^{-2}.

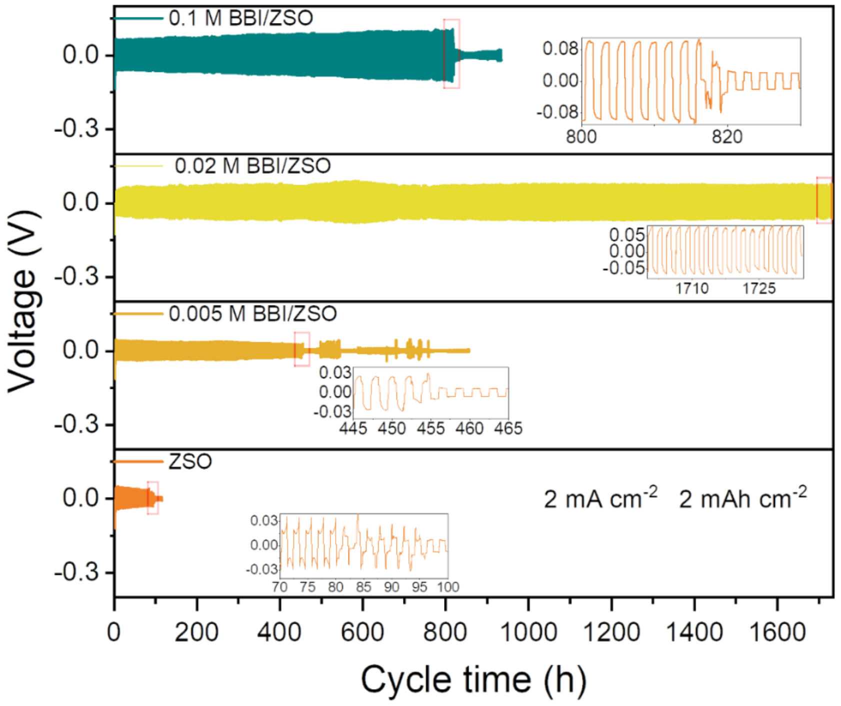

Figure S13. Long-term galvanostatic deposition/stripping of Zn||Zn symmetric cells tested in different BBI concentrations at 2 mA cm^{-2}@2 mA h cm^{-2}.

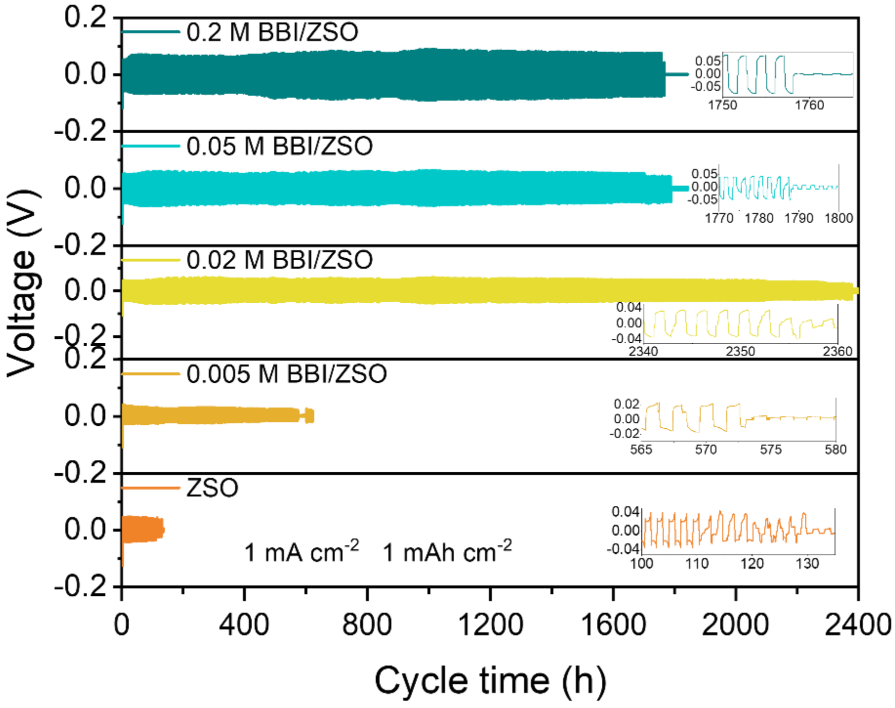

Figure S14. Long-term galvanostatic deposition/stripping of Zn||Zn symmetric cells tested in different BBI concentrations at 1 mA cm^{-2}@1 mA h cm^{-2}.

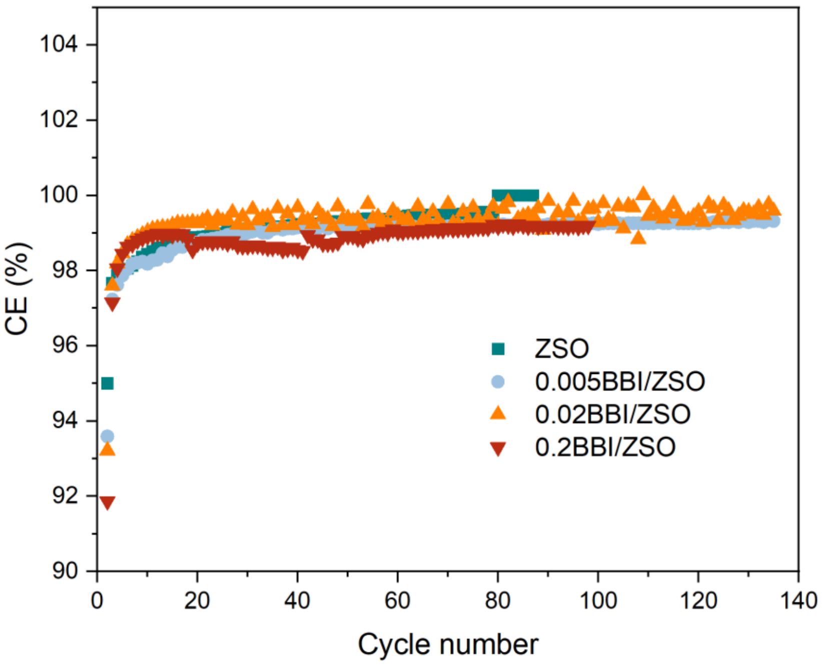

Figure S15. Comparison of Coulombic efficiency of different electrolytes.

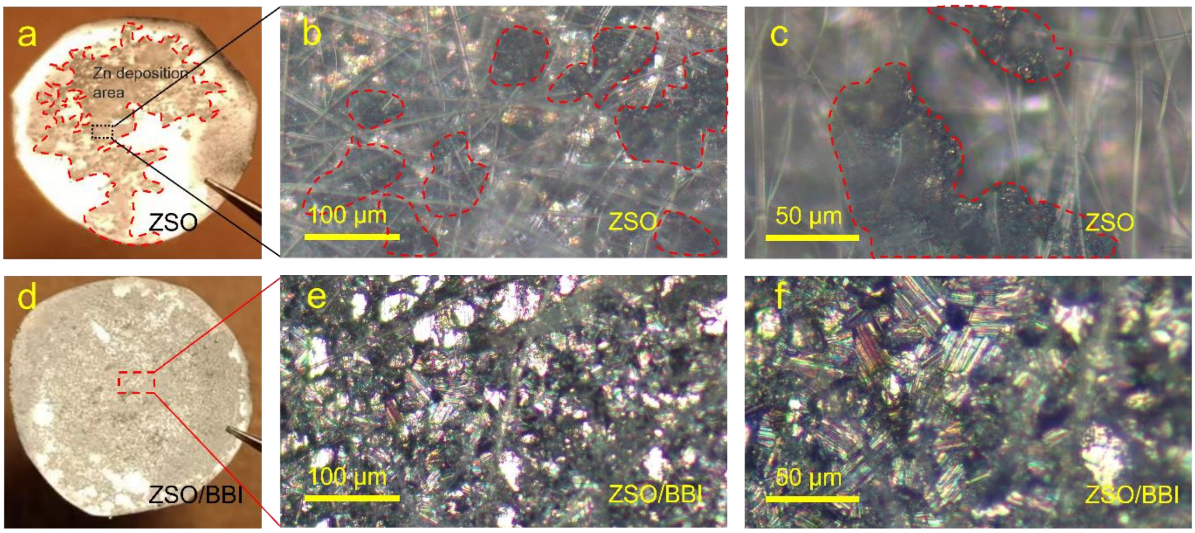

Figure S16. Optical images of Zn foil of Zn||Zn symmetric cells after 20 cycles under the current and capacity of 1 mA cm^{-2}@1 mA h cm^{-2} in ZSO electrolyte (a-c) and BBI/ZSO electrolyte (d-f).

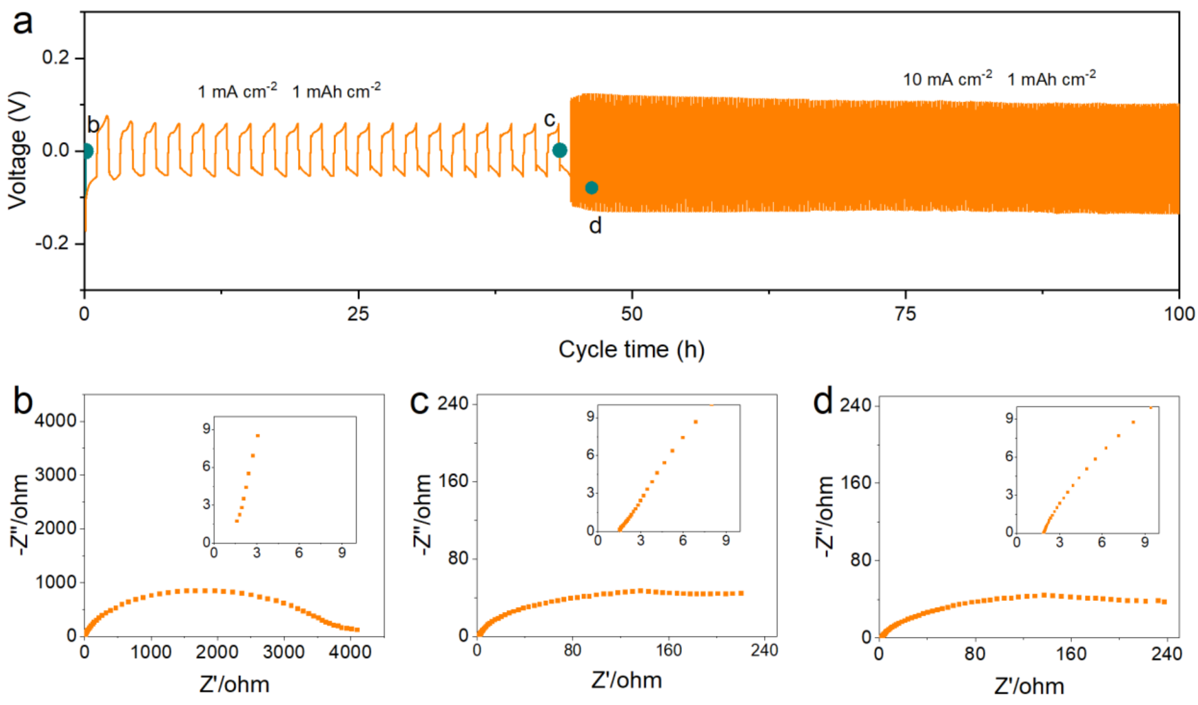

Figure S17. (a) The voltage profile of pre-cycles (BBI/ZSO) at 1 mA cm^{-2} and 1 mA h cm^{-2} followed by 10 mA cm^{-2} and 1 mA h cm^{-2}. The corresponding impedance spectra of the positions in (a): (b) before cycle, (c) after 20 cycles at 1 mA cm^{-2} and 1 mA h cm^{-2}, (d) after 10 cycles at 10 mA cm^{-2} and 1 mA h cm^{-2}.

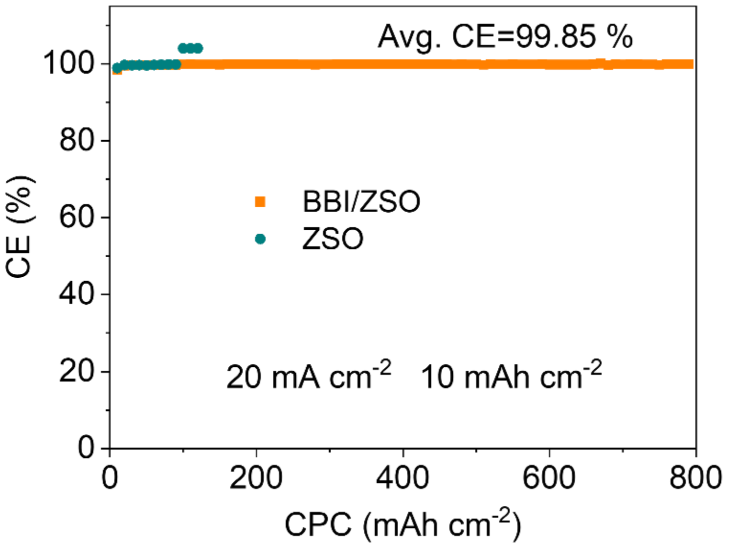

Figure S18. Coulombic efficiencies of Zn||Cu cells in ZSO and BBI/ZSO at 20 mA cm^{-2}@10 mA h cm^{-2}.

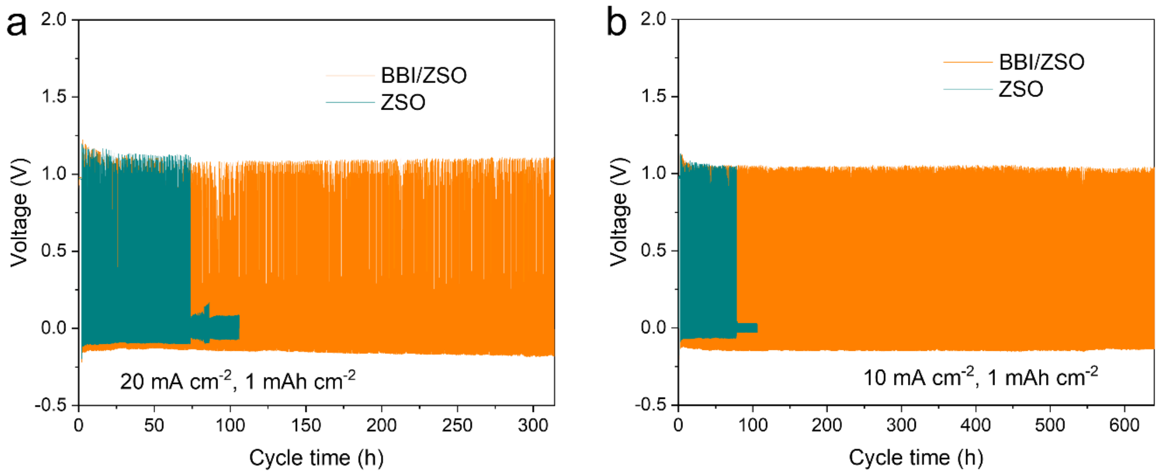

Figure S19. Voltage profiles of Zn||Cu cells in ZSO and BBI/ZSO electrolytes at 20 mA cm^{-2}@1 mA h cm^{-2} (a), and 10 mA cm^{-2}@1 mA h cm^{-2} (b).

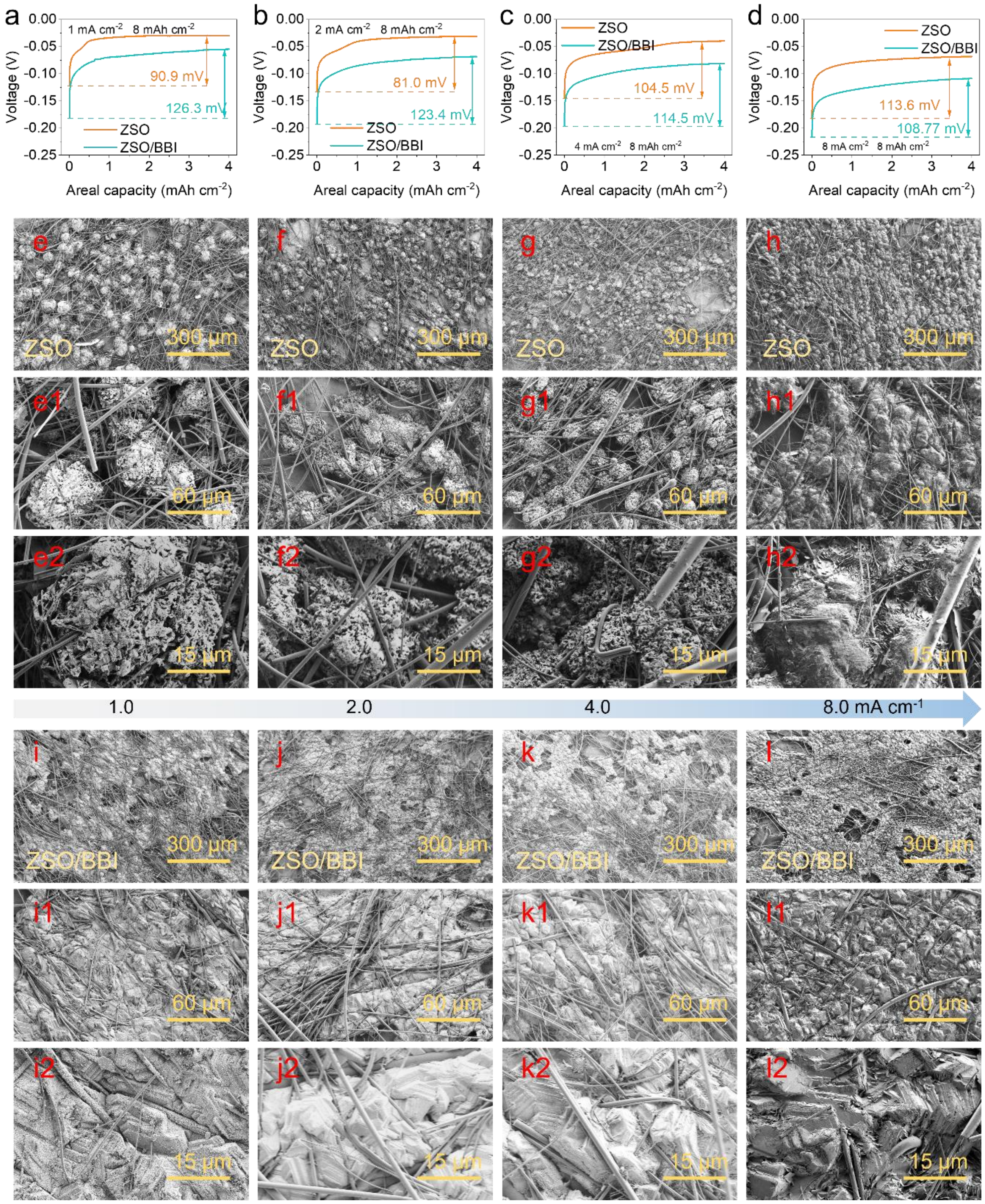

Figure S20. Capacity voltage profiles of the first nucleation of Zn in Zn||Zn symmetric cell at constant capacity 8 mA h cm^{-2}, but different currents, a. 1 mA cm^{-2}, b. 2 mA cm^{-2}, c. 4 mA cm^{-2}, d. 8 mA cm^{-2}. SEM images of deposition morphology of Zn with constant current but different capacities in electrolyte without (e-h) or with BBI (i-l). Figure e1 is an enlarged view of e, and Figure e2 is an enlarged view of e1. Other SEM images also have similar laws.

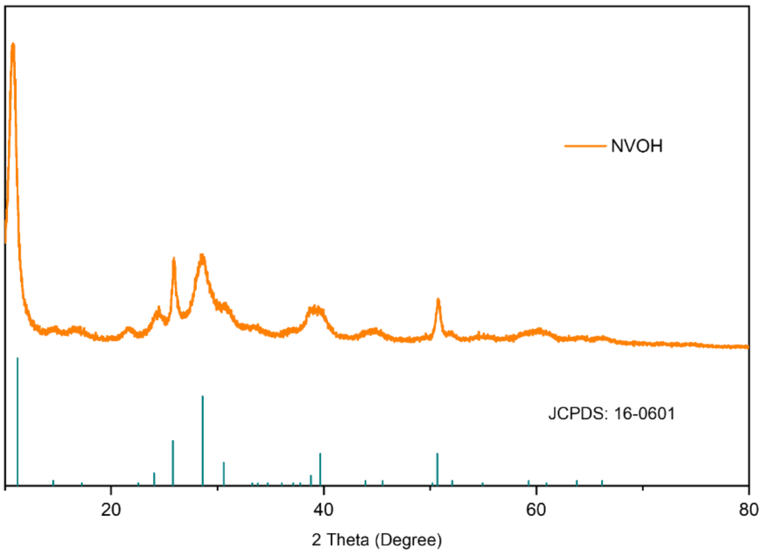

Figure S21. The XRD of NVOH cathode material.

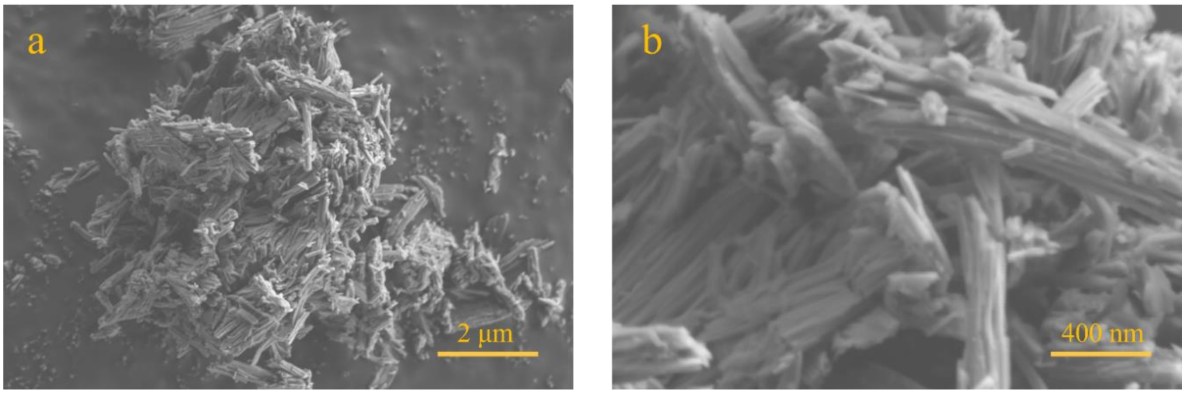

Figure S22. The SEM of NVOH cathode material.

Table S1. Cycling stability of Zn-Zn symmetric cells and Zn-based asymmetric cells between this work and previous research on additive optimization of electrolyte.

	Electrolyte formula	Current and capacity of Zn||Zn cells (mA-mAh cm^{-2})	Zn||Zn cells cycle time (h)	Current and capacity of asymmetric Zn||Cu cells (mA-mAh cm^{-2})	CE (%)	Ref.
Neutral additives	$Zn(ClO_4)_2$+10 mM β-cyclodextrin	1-1<br>5-5	1000<br>350	-	97.6%	Angew Chem Int Ed Engl 2022.[3]
	2 M $ZnSO_4$+1% gamma butyrolactone (GBL)	1-1<br>10-10<br>20-20	4200<br>1170<br>140	1-1	99.93%	Advanced Energy Materials 2022, 12 (38), 2202419.[4]
	1 M $ZnSO_4$ + Arginine(Arg)	1-1<br>5-4<br>10-4	2900<br>2200<br>900	1-1 (Zn||Ti)	98.26%	Advanced Functional Materials 2021, 31 (45), 2103514.[5]
	1 M $ZnSO_4$+5 mM thiourea (TU)	1-1<br>10-1	1200<br>600	10-1 (Zn||Ti)	98.9%	Advanced Functional Materials 2022.[6]
	2 M $ZnSO_4$+1% lithium magnesium silicate (LMS)	0.5-0.25<br>2.5-1.25	1000<br>400	2.5-1.25 (Zn||Ti)	98.5%	Nano Energy 2022, 93.[7]
	1 M $ZnSO_4$ + 0.5 wt % silk fibroin (SF)	1-1<br>3-3<br>10-5	1600<br>650<br>500	1-1	99.9%	ACS Nano 2022.[8]
	1 M $ZnSO_4$+ 10 mM glucose	1-1<br>5-5	2000<br>275	1-0.5 (Zn||Ti)	97.2%	Angew Chem Int Ed Engl 2021, 60, 18247.[9]
	1.2 m $Zn(ClO_4)_2$ +3.6 m methylsulfonylmethane (MSM)	1-0.25<br>2-0.5	2000<br>400	0.5-0.5	98.0%	Advanced Functional Materials 2022, 32.[10]
	2 M $ZnSO_4$+0.1 M 2-	1-1	1200	1-1 (Zn||Ti)	98.5%	Energy

	Bis(2-hydroxyethyl) amino-2(hydroxymethyl)-1,3-propanediol (BIS-TRIS)	5-5	600			Storage Materials 2021, 41, 515.[11]
Cationic additives	1 M $ZnSO_4$+4 M cholinium (Ch^+)	1-1<br>2-2	2000<br>800	0.5-0.5	99.6%	Advanced Functional Materials 2022, 32.[12]
	2 M $ZnSO_4$+0.05 M tetrabutylammonium sulfate (TBA_2SO_4)	2-2<br>5-5<br>10-2	300<br>160<br>400	10-10	98.0%	ACS Energy Letters 2020, 5, 3012.[13]
	2 M $ZnSO_4$+0.5 g L^{-1} benzyltrimethylammonium chloride (TMBAC)	0.5-1<br>5-5<br>10-5	900<br>500<br>450	1-1 (Zn||Ti)	99.0 %	Advanced Energy Materials 2022, 12.[14]
	0.5 M $ZnCl_2$+ 1 M triethylmethyl ammonium (TMA)	1-0.5<br>5-2.5	2145<br>500	1-0.5 (Zn||Ti)	98.5%	Advanced Energy Materials 2021, 12 (2), 2102780[15]
	0.5 M $ZnSO_4$+ 0.5 M TMA/H_2O	1-0.5	365	-	-	
Additives to assist SEI formation	2 m $ZnSO_4$+ 0.0085 m $La(NO_3)_3$	1-1<br>10-10	1200<br>160	2-1 (Zn||Ti)	99.9%	Nat Commun 2022, 13, 3252.[16]
	2 M $ZnSO_4$+0.05 M $LiPF_6$	2-4<br>5-10<br>10-20	1200<br>800<br>250	4-2 (Zn||Ti)	99.37%	Energy & Environmental Science 2021, 14, 3609.[17]
	1 M $Zn(OTF)_2$+ 25 × 10^{-3} M $Zn(H_2PO_4)_2$	1-1<br>1-5<br>5-1<br>5-5	1200<br>800<br>250<br>65	0.5-0.5	99.4%	Adv Mater 2021, 33, e2007416.[18]
	4 m $Zn(OTF)_2$ + 0.5 m Trimethylethyl ammonium trifluoromethanesulfonate ($Me_3EtNOTF$)	0.5-0.25	6000	0.5-0.5	99.9%	Nat Nanotechnol 2021, 16, 902.[19]
	3 M $Zn(OTF)_2$+20 mM $Zn(NO_3)_2$	0.5-0.5	1200	1-0.5 (Zn||Ti)	99.8%	Angew Chem Int Ed Engl 2021, 60, 13035.[20]
Anionic	1 M $ZnSO_4$+0.01 M	1-1	4400	1-1	99.72%	Energy &

additives	Sodium 3,3-dithiodipropane sulfonate	5-5	870			Environmental Science 2023, 16 (2), 687-697.[21]
	2 M $ZnSO_4$+0.5 g L^{-1} saccharin (Sac)	2-2<br>10-10<br>40-10	600<br>500<br>220	10-10	99.6%	Adv Mater 2021, 33, e2100445.[22]
	1 M $Zn(BBI)_2$<br>Zinc bis (benzenesulfonyl)imide	2-2<br>5-5<br>20-1	2800<br>1000<br>700	5-5	99.7%	Adv Mater 2023, e2210055[23]
	1 m $ZnSO_4$+0.02 m BBI	1-1<br>2-2<br>5-1<br>5-5<br>10-1<br>10-10<br>20-1<br>30-1	2350<br>1735<br>3630<br>1180<br>1280<br>190<br>940<br>470	2-1<br>10-1<br>10-5<br>20-10	99.7%<br>99.88%<br>99.83%<br>99.85%	**This work**

References

[1] C. J. Powell, A. Jablonski, F. Salvat, *Surface and Interface Analysis* **2005**, 37, 1068.

[2] Z. Cai, J. Wang, Z. Lu, R. Zhan, Y. Ou, L. Wang, M. Dahbi, J. Alami, J. Lu, K. Amine, Y. Sun, *Angew. Chem. Int. Ed. Engl.* **2022**, 61, e202116560.

[3] M. Qiu, P. Sun, Y. Wang, L. Ma, C. Zhi, W. Mai, *Angew. Chem. Int. Ed. Engl.* **2022**, 61, e202210979.

[4] H. Huang, D. Xie, J. Zhao, P. Rao, W. M. Choi, K. Davey, J. Mao, *Adv. Energy Mater.* **2022**, 12, 2202419.

[5] H. Lu, X. Zhang, M. Luo, K. Cao, Y. Lu, B. B. Xu, H. Pan, K. Tao, Y. Jiang, *Adv. Funct. Mater.* **2021**, 31, 2103514.

[6] H. Qin, W. Kuang, N. Hu, X. Zhong, D. Huang, F. Shen, Z. Wei, Y. Huang, J. Xu, H. He, *Adv. Funct. Mater.* **2022**, 32, 2206695.

[7] J. Cao, D. Zhang, Y. Yue, R. Chanajaree, S. Wang, J. Han, X. Zhang, J. Qin, Y. Huang, *Nano Energy* **2022**, 93, 106839.

[8] J. Xu, W. Lv, W. Yang, Y. Jin, Q. Jin, B. Sun, Z. Zhang, T. Wang, L. Zheng, X. Shi, B. Sun, G. Wang, *ACS Nano* **2022**, 16, 11392.

[9] P. Sun, L. Ma, W. Zhou, M. Qiu, Z. Wang, D. Chao, W. Mai, *Angew. Chem. Int. Ed. Engl.* **2021**, 60, 18247.

[10] M. Han, J. Huang, X. Xie, T. C. Li, J. Huang, S. Liang, J. Zhou, H. J. Fan, *Adv. Funct. Mater.* **2022**, 32, 2110957.

[11] M. Luo, C. Wang, H. Lu, Y. Lu, B. B. Xu, W. Sun, H. Pan, M. Yan, Y. Jiang, *Energy Stor. Mater.* **2021**, 41, 515.

[12] X. Nie, L. Miao, W. Yuan, G. Ma, S. Di, Y. Wang, S. Shen, N. Zhang, *Adv. Funct. Mater.* **2022**, 32, 2203905.

[13] A. Bayaguud, X. Luo, Y. Fu, C. Zhu, *ACS Energy Lett.* **2020**, 5, 3012.

[14] K. Guan, L. Tao, R. Yang, H. Zhang, N. Wang, H. Wan, J. Cui, J. Zhang, H. Wang, H. Wang, *Adv. Energy Mater.* **2022**, 12, 2103557.

[15] R. Yao, L. Qian, Y. Sui, G. Zhao, R. Guo, S. Hu, P. Liu, H. Zhu, F. Wang, C. Zhi, C. Yang, *Adv. Energy Mater.* **2021**, 12, 2102780.

[16] R. Zhao, H. Wang, H. Du, Y. Yang, Z. Gao, L. Qie, Y. Huang, *Nat Commun* **2022**, 13, 3252.

[17] Y. Chu, S. Zhang, S. Wu, Z. Hu, G. Cui, J. Luo, *Energy & Environmental Science* **2021**, 14, 3609.

[18] X. Zeng, J. Mao, J. Hao, J. Liu, S. Liu, Z. Wang, Y. Wang, S. Zhang, T. Zheng, J. Liu, P. Rao, Z. Guo, *Adv Mater* **2021**, 33, e2007416.

[19] L. Cao, D. Li, T. Pollard, T. Deng, B. Zhang, C. Yang, L. Chen, J. Vatamanu, E. Hu, M. J. Hourwitz, L. Ma, M. Ding, Q. Li, S. Hou, K. Gaskell, J. T. Fourkas, X. Q. Yang, K. Xu, O. Borodin, C. Wang, *Nat. Nanotechnol.* **2021**, 16, 902.

[20] D. Li, L. Cao, T. Deng, S. Liu, C. Wang, *Angew Chem Int Ed Engl* **2021**, 60, 13035.

[21] Y. Lin, Z. Mai, H. Liang, Y. Li, G. Yang, C. Wang, *Energy Environ. Sci.* **2023**, 16, 687.

[22] C. Huang, X. Zhao, S. Liu, Y. Hao, Q. Tang, A. Hu, Z. Liu, X. Chen, *Adv. Mater.* **2021**, 33, e2100445.

[23] H. Du, Y. Dong, Q. J. Li, R. Zhao, X. Qi, W. H. Kan, L. Suo, L. Qie, J. Li, Y. Huang, *Adv. Mater.* **2023**, e2210055.
